# Supplementary material for: Reproducibility of real-world evidence studies using clinical practice data to inform regulatory and coverage decisions
Source: Nat Commun. 2022 Aug 31;13:5126. doi: 10.1038/s41467-022-32310-3 (PMC9430007; doi:10.1038/s41467-022-32310-3)
Supplement: Supplementary file 1 — Supplementary Information [file 41467_2022_32310_MOESM1_ESM.docx]

SUPPLEMENTARY INFORMATION FILE

Title: Reproducibility of real-world evidence studies using clinical practice data to inform regulatory and coverage decisions

**Authors:**

Shirley Wang, Sushama Kattinakere Sreedhara and Sebastian Schneeweiss

on behalf of the REPEAT Initiative^1^

**^1^Contributors and Affiliations**

* indicates co-author

† indicates acknowledged for contributions

**Principal investigators:**

Shirley V Wang PhD, ScM*

Division of Pharmacoepidemiology and Pharmacoeconomics, Department of Medicine, Brigham and Women’s Hospital, Boston, Massachusetts

Associate Professor of Medicine, Harvard Medical School, Boston, Massachusetts

Sebastian Schneeweiss MD, ScD*

Division of Pharmacoepidemiology and Pharmacoeconomics, Department of Medicine, Brigham and Women’s Hospital, Boston, Massachusetts

Professor of Medicine, Harvard Medical School, Boston, Massachusetts

Professor of Epidemiology, Harvard Chan School of Public Health

**Co-investigator Faculty (in alphabetical order):**

Jessica M Franklin, PhD^1*^

Division of Pharmacoepidemiology and Pharmacoeconomics, Department of Medicine, Brigham and Women’s Hospital, Harvard Medical School, Boston, Massachusetts

Joshua J Gagne, PharmD, ScD^2*^

Division of Pharmacoepidemiology and Pharmacoeconomics, Department of Medicine, Brigham and Women’s Hospital, Harvard Medical School, Boston, Massachusetts

Krista F Huybrechts, PhD, MS^*^

Division of Pharmacoepidemiology and Pharmacoeconomics, Department of Medicine, Brigham and Women’s Hospital, Harvard Medical School, Boston, Massachusetts

Elisabetta Patorno, MD, DrPh^*^

Division of Pharmacoepidemiology and Pharmacoeconomics, Department of Medicine, Brigham and Women’s Hospital, Harvard Medical School, Boston, Massachusetts

**Reproduction Team (in alphabetical order):**

*Lead Research Specialist*

Sushama Kattinakere Sreedhara MBBS, MSPH^*^

Division of Pharmacoepidemiology and Pharmacoeconomics, Department of Medicine, Brigham and Women’s Hospital, Boston, Massachusetts

*Research Specialists*

Yinzhu Jin MS, MPH^*^

Division of Pharmacoepidemiology and Pharmacoeconomics, Department of Medicine, Brigham and Women’s Hospital, Boston, Massachusetts

Moa Lee PharmD, MPH^*^

Division of Pharmacoepidemiology and Pharmacoeconomics, Department of Medicine, Brigham and Women’s Hospital, Boston, Massachusetts

Mufaddal Mahesri MD, MPH^*^

Division of Pharmacoepidemiology and Pharmacoeconomics, Department of Medicine, Brigham and Women’s Hospital, Boston, Massachusetts

Ajinkya Pawar PhD, MS^*^

Division of Pharmacoepidemiology and Pharmacoeconomics, Department of Medicine, Brigham and Women’s Hospital, Boston, Massachusetts

*Research Assistants*

Julie Barberio BS^*^

Division of Pharmacoepidemiology and Pharmacoeconomics, Department of Medicine, Brigham and Women’s Hospital, Boston, Massachusetts

Lily G Bessette BS^*^

Division of Pharmacoepidemiology and Pharmacoeconomics, Department of Medicine, Brigham and Women’s Hospital, Boston, Massachusetts

Kristyn Chin BS^*^

Division of Pharmacoepidemiology and Pharmacoeconomics, Department of Medicine, Brigham and Women’s Hospital, Boston, Massachusetts

Nileesa Gautam BS^*^

Division of Pharmacoepidemiology and Pharmacoeconomics, Department of Medicine, Brigham and Women’s Hospital, Boston, Massachusetts

Adrian Santiago Ortiz BS^*^

Division of Pharmacoepidemiology and Pharmacoeconomics, Department of Medicine, Brigham and Women’s Hospital, Boston, Massachusetts

Ellen Sears BS^*^

Division of Pharmacoepidemiology and Pharmacoeconomics, Department of Medicine, Brigham and Women’s Hospital, Boston, Massachusetts

Kristina Stefanini BA^*^

Division of Pharmacoepidemiology and Pharmacoeconomics, Department of Medicine, Brigham and Women’s Hospital, Boston, Massachusetts

Mimi Zakarian BS^*^

Division of Pharmacoepidemiology and Pharmacoeconomics, Department of Medicine, Brigham and Women’s Hospital, Boston, Massachusetts

*Research Analysts*

Sara Dejene BS^*^

Division of Pharmacoepidemiology and Pharmacoeconomics, Department of Medicine, Brigham and Women’s Hospital, Boston, Massachusetts

James R Rogers, MS^*^

Division of Pharmacoepidemiology and Pharmacoeconomics, Department of Medicine, Brigham and Women’s Hospital, Boston, Massachusetts

*Statistical Programmers*

Gregory Brill^*^

Division of Pharmacoepidemiology and Pharmacoeconomics, Department of Medicine, Brigham and Women’s Hospital, Boston, Massachusetts

Joan Landon MPH^*^

Division of Pharmacoepidemiology and Pharmacoeconomics, Department of Medicine, Brigham and Women’s Hospital, Boston, Massachusett^s^

Joyce Lii MS MA*

Division of Pharmacoepidemiology and Pharmacoeconomics, Department of Medicine, Brigham and Women’s Hospital, Boston, Massachusetts

Ted Tsacogianis MPH^*^

Division of Pharmacoepidemiology and Pharmacoeconomics, Department of Medicine, Brigham and Women’s Hospital, Boston, Massachusetts

Seanna Vine MPH^*^

Division of Pharmacoepidemiology and Pharmacoeconomics, Department of Medicine, Brigham and Women’s Hospital, Boston, Massachusetts

*Science and Implementation Specialists*

Elizabeth M Garry PhD, MPH^*^

Aetion, Inc., New York, New York

Liza R Gibbs, BS^*^

Aetion, Inc., New York, New York

Monica Gierada MPH^*^

Aetion, Inc., New York, New York

Danielle L Isaman, MS^*^

Aetion, Inc., New York, New York

Emma Payne BS^*^

Aetion, Inc., New York, New York

**Scientific Advisory Board (in alphabetical order):**

Sarah Alwardt PhD*

McKesson Specialty Health, Irving, Texas

Peter Arlett BSc MBBS MRCP FFPM†

Pharmacovigilance and Epidemiology Department, European Medicines Agency, Amsterdam, Netherlands

Dorothee B. Bartels MSc PhD*

UCB Pharma, Germany

Andrew Bate PhD*

GSK, Middlesex United Kingdom

Jesse Berlin ScD†

Johnson & Johnson, New Brunswick, New Jersey

Alison Bourke MSc FRPharmS*

IQVIA, London, United Kingdom

Brian Bradbury DSc†

Center for Observational Research, Amgen Inc., Thousand Oaks, California

Karen Burnett MBA MS†

Northwestern Memorial Hospital, Chicago Il

Troyen Brennan MD†

CVS Health, Woonsocket, Rhode Island

Jeffrey Brown PhD*

Department of Population Medicine, Harvard Medical School, Boston MA

K. Arnold Chan MD ScD*

Health Data Research Center, National Taiwan University, Taipei, Taiwan

Nam-Kyong Choi B. Pharm PhD*

Department of Health Convergence, Ewha Womans University, Seoul, South Korea

Frank de Vries PharmD PhD†

Department of Clinical Pharmacy, Maastricht University Medical Center+, Maastricht, Netherlands

Kristian B. Filion PhD*

Department of Medicine and Department of Epidemiology, Biostatistics, and Occupational Health, McGill University, Montreal, Canada

Centre for Clinical Epidemiology, Lady Davis Institute, Jewish General Hospital, Montreal, Canada.

Lisa Freeman†

Connecticut Center for Patient Safety, LLC, Fairfield, Connecticut

Hans-Georg Eichler MD, MSc†

European Medicines Agency, Amsterdam, Netherlands and Medical University of Vienna, Vienna, Austria

Jesper Hallas MD PhD*

University of Southern Denmark, Odense, Denmark

Laura Happe PharmD MPH*
Department of Pharmaceutical Outcomes and Policy, University of Florida College of Pharmacy, Gainesville, Florida

Sean Hennessy PharmD, PhD*

Perelman School of Medicine, University of Pennsylvania, Philadelphia, PA

John Ioannidis MD DSc†

Meta-Research Innovation Center at Stanford, Stanford University, Stanford, California

Javier Jimenez MD MPH†

Real-World Evidence and Clinical Outcomes, Sanofi S.A., Paris, France

Páll Jónsson PhD Mres*

National Institute for Health and Care Excellence, London, United Kingdom

Kristijan H Kahler PhD*

Evidence & Launch Excellence, Novartis Pharmaceuticals Corporation, East Hanover NJ, USA

Christine Laine MD MPH FACP†

Annals of Internal Medicine, Philadelphia, Pennsylvania

Elizabeth Loder MD MPH†

The BMJ, London, United Kingdom

Amr Makady PharmD PhD*

Janssen-Cilag B.V., Breda, Netherlands

David Martin MD MPH*^3^

Food and Drug Administration, Silver Spring, Maryland

Michael Nguyen MD*

Food and Drug Administration, Silver Spring, Maryland

Brian Nosek PhD*

Center for Open Science, Charlottesville, Virginia

Richard Platt MD MSc†

Department of Population Medicine, Harvard Medical School, Boston MA

Robert W Platt PhD†

Department of Epidemiology, Biostatistics, and Occupational Health and Department of Pediatrics, McGill University, Montreal, Canada

John Seeger PharmD DrPh*

Optum Epidemiology, Optum, Eden Prairie, Minnesota

William Shrank MD*

Humana, Washington DC

Liam Smeeth PhD*

London School of Hygiene and Tropical Medicine, University of London, London, United Kingdom

Henrik Toft Sørensen MD PhD DMSc DSc*

Aarhus University, Denmark

Peter Tugwell MSc MD FRCPC*

Journal of Clinical Epidemiology, Ottawa, Canada

Yoshiaki Uyama PhD†

Pharmaceuticals and Medical Devices Agency, Tokyo, Japan

Richard Willke PhD*

The International Society for Health Economics and Outcomes Research, Lawrenceville, New Jersey

Wolfgang Winkelmayer MD MPH ScD FASN*

Baylor College of Medicine, Houston, TX

Deborah Zarin†

Division of Global Health Equity, Department of Medicine, Brigham and Women’s Hospital, Harvard Medical School, Boston, Massachusetts

^1^ During the conduct of the project, Dr. Franklin was faculty at the Division of Pharmacoepidemiology and Pharmacoeconomics at Brigham and Women’s Hospital, Harvard Medical School. She is now employed by Optum Epidemiology.

^2^ During the conduct of the project, Dr. Gagne was faculty at the Division of Pharmacoepidemiology and Pharmacoeconomics at Brigham and Women’s Hospital, Harvard Medical School. He is now employed by Johnson & Johnson.

^3^ During the conduct of this work, Dr. Martin was employed by the FDA. He is now employed by Moderna.

**Supplementary Figure 1. PRISMA Diagram**


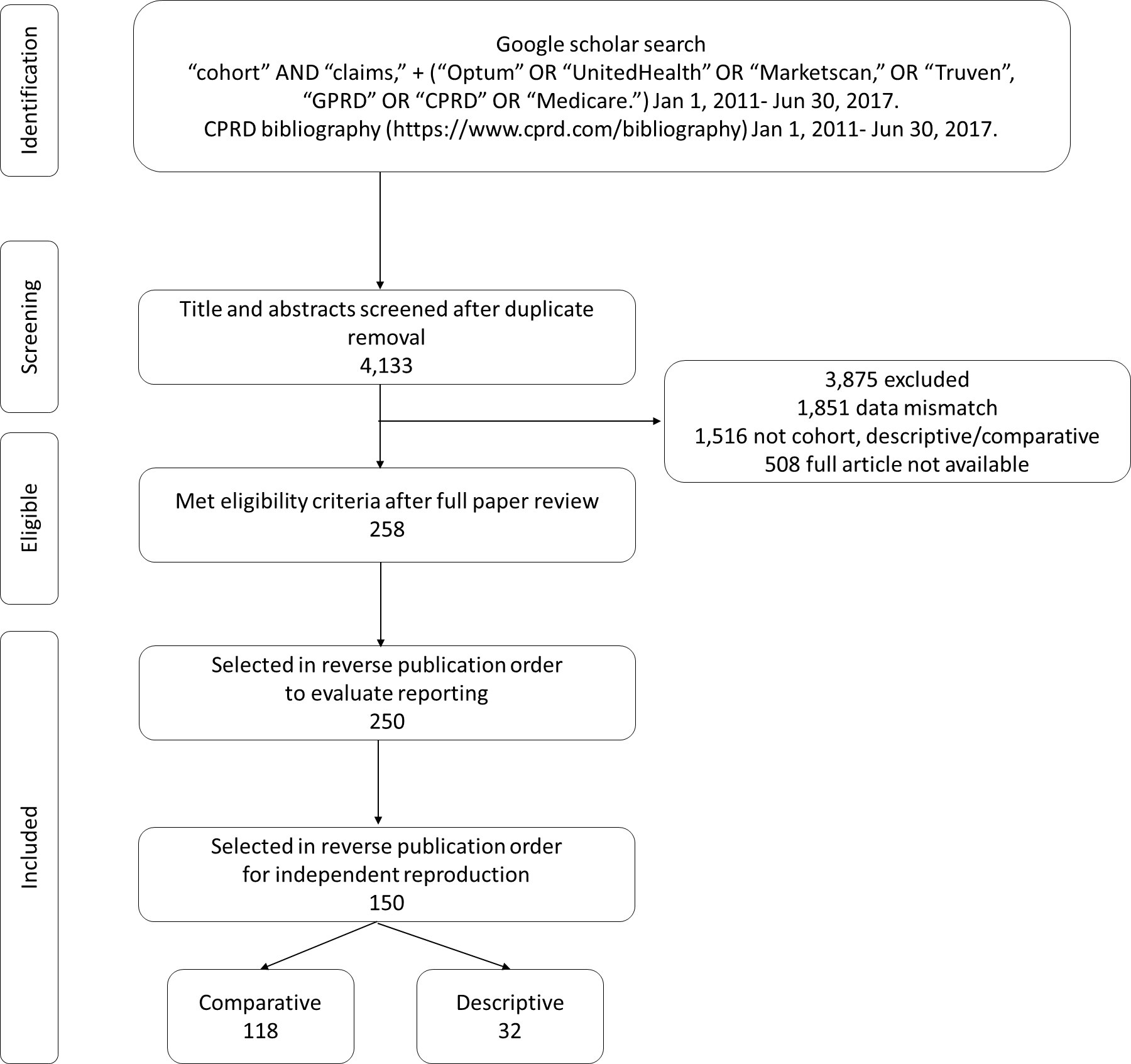


**Supplementary Figure 2 Stratified differences in prevalence of baseline characteristics (original - reproduction)**

**A. Stratified by study type - comparative versus descriptive**


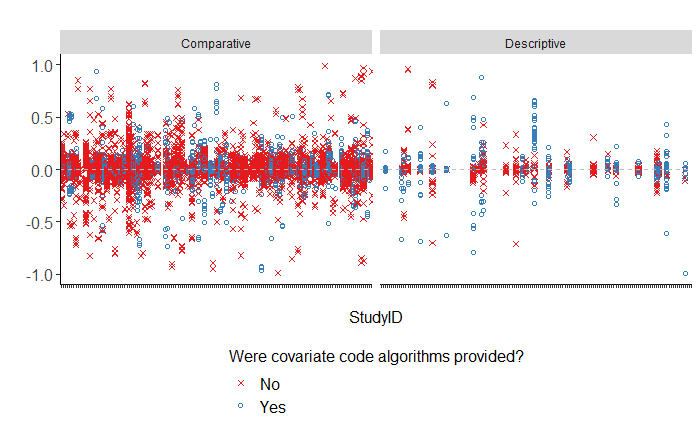


**B. Stratified by relative study size of original versus reproduction**


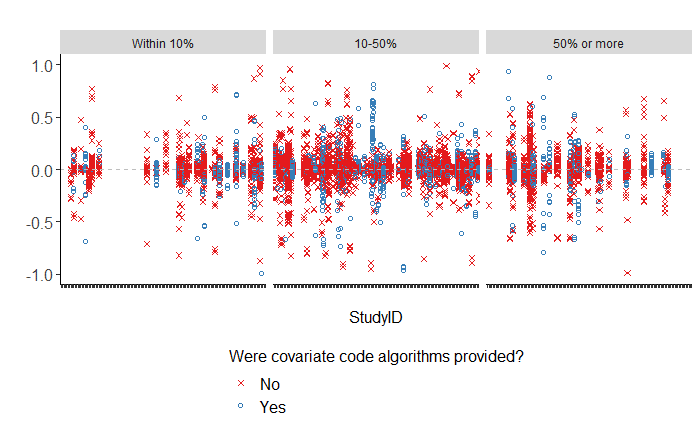

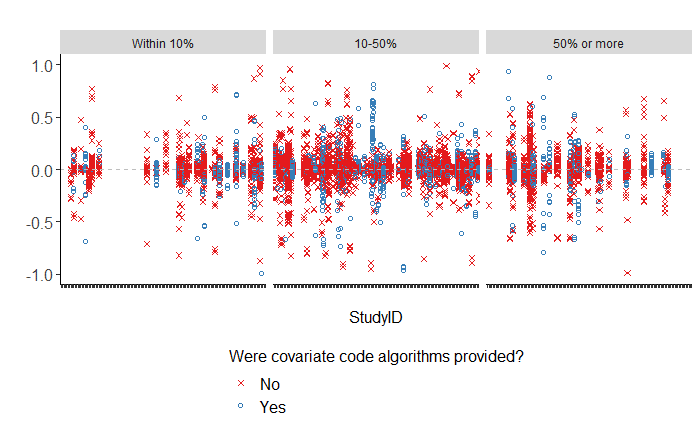


Horizontal dashed gray lines at 0.0 indicate where there is no difference in prevalence of a baseline characteristic between the original cohort and the reproduction. Source data are provided as a Source Data file.

**Supplementary Figure 3 Distribution of relative magnitude of hazard ratio, risk ratio, odds ratio (original/reproduction) within strata defined by how closely the relative sample size was reproduced**


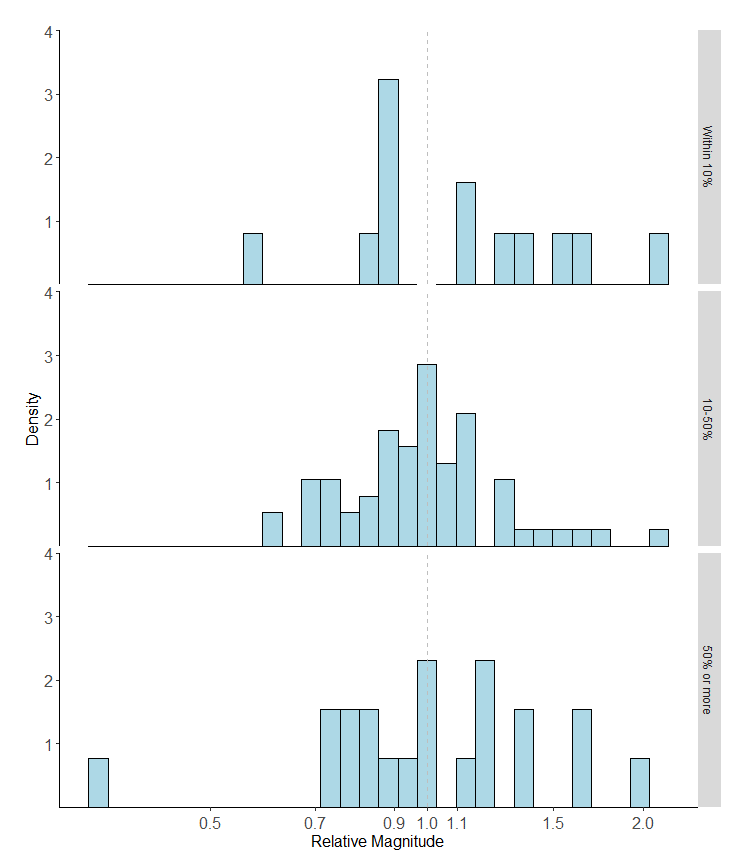


Vertical dashed gray line indicates where the effect size of the original and the reproduction are equal. Source data are provided as a Source Data file.

**Supplementary Figure 4. Bland-Altman plot showing the relationship between magnitude of risks, rates and reproducibility (difference between original and reproduced coefficients)**

**A. Risks**


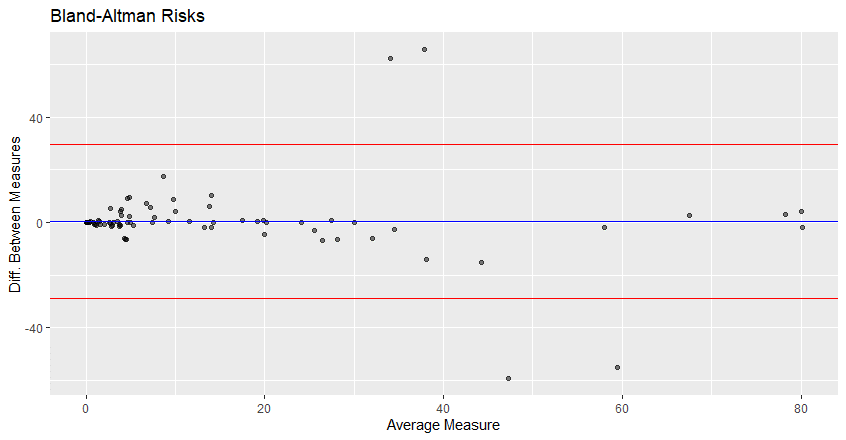


**B. Rates**


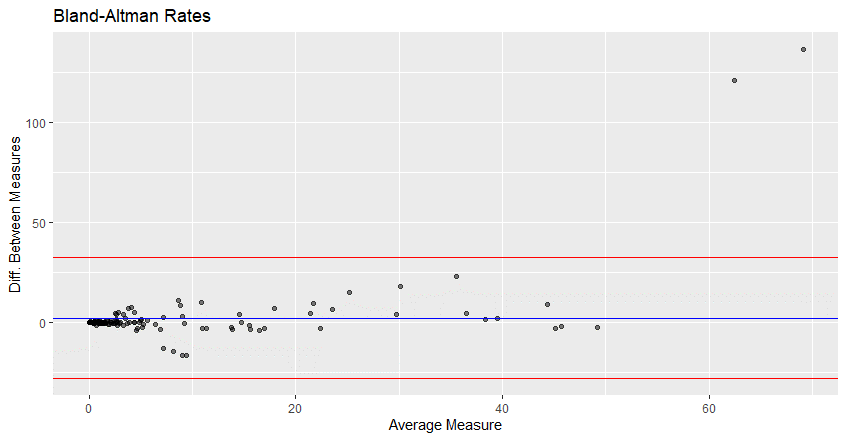


The blue horizontal line marks the average difference in log effect size between the original and reproduction. The red horizontal lines mark ±2 times the standard deviation of the difference in log effect size. Source data are provided as a Source Data file.

**Supplementary Materials Table 1. Clarity of reporting and other study characteristics in a sample of 250 real-world evidence studies**

|  |  | **Overall** | | **Descriptive  Studies** | | **Comparative Studies** | |  |  |  |  |
| --- | --- | --- | --- | --- | --- | --- | --- | --- | --- | --- | --- |
|  |  | **N = 250** | | **N = 89** | | **N = 161** | |  |  |  |  |
| **Characteristic** | **Category** | **N** | **%** | **N** | **%** | **N** | **%** |  |  |  |  |
|  |  |  |  |  |  |  |  |  |  |  |  |
|  |  |  |  |  |  |  |  |  |  |  |  |
| Data Source |  |  |  |  |  |  |  |  | datasource |  |  |
|  | CPRD | 18 | 7.2 | 5 | 5.6 | 13 | 8.1 |  |  |  |  |
|  | MarketScan | 109 | 43.6 | 32 | 36.0 | 77 | 47.8 |  |  |  |  |
|  | Medicare | 99 | 39.6 | 47 | 52.8 | 52 | 32.3 |  |  |  |  |
|  | Optum | 24 | 9.6 | 5 | 5.6 | 19 | 11.8 |  |  |  |  |
| Were the years of source data available for the study specified? |  |  |  |  |  |  |  |  | source_data_range |  |  |
|  | No | 7 | 2.8 | 1 | 1.1 | 6 | 3.7 |  |  |  |  |
|  | Yes | 243 | 97.2 | 88 | 98.9 | 155 | 96.3 |  |  |  |  |
| Was source data converted to a common data model? |  |  |  |  |  |  |  |  | sourcedata_cdm |  |  |
|  | No | 245 | 98.0 | 89 | 100.0 | 156 | 96.9 |  |  |  |  |
|  | Yes | 5 | 2.0 |  |  | 5 | 3.1 |  |  |  |  |
| Was a design diagram provided? |  |  |  |  |  |  |  |  | design_diagram |  |  |
|  | No | 231 | 92.4 | 86 | 96.6 | 145 | 90.1 |  |  |  |  |
|  | Yes | 19 | 7.6 | 3 | 3.4 | 16 | 9.9 |  |  |  |  |
| Was an attrition table provided? |  |  |  |  |  |  |  |  | attrition_table |  |  |
|  | No | 114 | 45.6 | 54 | 60.7 | 60 | 37.3 |  |  |  |  |
|  | Yes | 136 | 54.4 | 35 | 39.3 | 101 | 62.7 |  |  |  |  |
| Did the authors specify how missing data or out of range values were handled? |  |  |  |  |  |  |  |  | missing_data_method |  |  |
|  | No | 211 | 84.4 | 80 | 89.9 | 131 | 81.4 |  |  |  |  |
|  | Yes | 39 | 15.6 | 9 | 10.1 | 30 | 18.6 |  |  |  |  |
| Did the authors clearly define the study entry date? |  |  |  |  |  |  |  |  | index_date |  |  |
|  | No | 27 | 10.8 | 15 | 16.9 | 12 | 7.5 |  |  |  |  |
|  | Yes | 223 | 89.2 | 74 | 83.1 | 149 | 92.5 |  |  |  |  |
| Did the authors specify how many times an individual could enter the study population? |  |  |  |  |  |  |  |  | number_entries |  |  |
|  | No, not defined. | 77 | 30.8 | 27 | 30.3 | 50 | 31.1 |  |  |  |  |
|  | Yes, every time entry criteria are met. | 24 | 9.6 | 16 | 18.0 | 8 | 5.0 |  |  |  |  |
|  | Yes, first time entry criteria are met only. | 149 | 59.6 | 46 | 51.7 | 103 | 64.0 |  |  |  |  |
| Did the authors clearly define the timing of required observable time prior to the study entry date? |  |  |  |  |  |  |  |  | enrollment_window |  |  |
|  | No | 46 | 18.4 | 21 | 23.6 | 25 | 15.5 |  |  |  |  |
|  | Yes | 204 | 81.6 | 68 | 76.4 | 136 | 84.5 |  |  |  |  |
| Did the authors report whether (and how) coverage gaps in observable time were bridged? |  |  |  |  |  |  |  |  | enrollment_coverage_rc |  |  |
|  | No | 243 | 97.2 | 86 | 96.6 | 157 | 97.5 |  |  |  |  |
|  | Yes | 7 | 2.8 | 3 | 3.4 | 4 | 2.5 |  |  |  |  |
| Did the authors clearly define the time window during which inclusion-exclusion criteria are assessed relative to the study entry date? |  |  |  |  |  |  |  |  | inclusion_exclusion_window |  |  |
|  | No | 49 | 19.6 | 10 | 11.2 | 39 | 24.2 |  |  |  |  |
|  | Yes | 191 | 76.0 | 70 | 78.7 | 120 | 74.5 |  |  |  |  |
|  | n/a | 10 | 4.4 | 9 | 10.1 | 2 | 1.2 |  |  |  |  |
| Did the authors specify when exclusion criteria are applied relative to selection of the study entry date? |  |  |  |  |  |  |  |  | order_exclusions_re_index |  |  |
|  | All potential cohort entry dates were identified for each individual, then exclusion criteria applied. Every cohort entry date that met all inclusion/exclusion criteria was included (multiple entries | 21 | 8.4 | 12 | 13.5 | 9 | 5.6 |  |  |  |  |
|  | All potential cohort entry dates were identified for each individual, then exclusion criteria applied. The first cohort entry date for each individual that met all inclusion/exclusion criteria was inc | 37 | 14.8 | 9 | 10.1 | 28 | 17.4 |  |  |  |  |
|  | All potential cohort entry dates were identified for each individual, then exclusion criteria applied. The first cohort entry date that met criteria was kept. (e.g. multiple "incident user"episodes) | 5 | 2.0 | 2 | 2.2 | 3 | 1.9 |  |  |  |  |
|  | No, not defined. | 60 | 24.0 | 21 | 23.6 | 39 | 24.2 |  |  |  |  |
|  | The first cohort entry date for each patient was identified, then exclusion criteria applied. If criteria not met, potential cohort entry dates at a later time were not evaluated. | 114 | 45.6 | 36 | 40.4 | 78 | 48.4 |  |  |  |  |
|  | n/a | 13 | 5.2 | 9 | 10.1 | 4 | 2.5 |  |  |  |  |
| Did the authors specify the type of exposure being measured? |  |  |  |  |  |  |  |  | exposure_type |  |  |
|  | incident | 90 | 36.0 | - | - | 90 | 55.9 |  |  |  |  |
|  | cumulative | 1 | 0.4 | - | - | 1 | 0.6 |  |  |  |  |
|  | prevalent | 14 | 5.6 | - | - | 14 | 8.7 |  |  |  |  |
|  | time-varying | 9 | 3.6 | - | - | 9 | 5.6 |  |  |  |  |
|  | other or not defined | 44 | 17.6 | - | - | 44 | 27.3 |  |  |  |  |
|  | n/a | 92 | 36.8 | 89 | 100.0 | 3 | 1.9 |  |  |  |  |
| Did the authors clearly define the washout window for incident exposures relative to the study entry date? |  |  |  |  |  |  |  |  | washout_exposure |  |  |
|  | No | 33 | 13.2 | - | - | 33 | 20.5 |  |  |  |  |
|  | Yes | 81 | 32.4 | - | - | 81 | 50.3 |  |  |  |  |
|  | n/a | 136 | 54.4 | 89 | 100.0 | 47 | 29.2 |  |  |  |  |
| Did the authors address how early refills are handled when defining exposure duration? |  |  |  |  |  |  |  |  | early_refills |  |  |
|  | No | 72 | 28.8 | - | - | 72 | 44.7 |  |  |  |  |
|  | Yes | 6 | 2.4 | - | - | 6 | 3.7 |  |  |  |  |
|  | n/a | 172 | 68.8 | 89 | 100.0 | 83 | 51.6 |  |  |  |  |
| Did the authors address how gaps in days supply between consecutive dispensations or prescriptions are handled when defining exposure duration? |  |  |  |  |  |  |  |  | exposure_gap |  |  |
|  | No | 33 | 13.2 | - | - | 33 | 20.5 |  |  |  |  |
|  | Yes | 44 | 17.6 | - | - | 44 | 27.3 |  |  |  |  |
|  | n/a | 173 | 69.2 | 89 | 100.0 | 84 | 52.2 |  |  |  |  |
| Did the authors address how extension of hypothesized effect of exposure effect the last days supply was handled when defining exposure duration? |  |  |  |  |  |  |  |  | exposure_extension |  |  |
|  | No | 34 | 13.6 | - | - | 34 | 21.1 |  |  |  |  |
|  | Yes | 42 | 16.8 | - | - | 42 | 26.1 |  |  |  |  |
|  | n/a | 174 | 69.6 | 89 | 100.0 | 85 | 52.8 |  |  |  |  |
| Did the authors clearly define the washout window for incident outcomes relative to the study entry date? |  |  |  |  |  |  |  |  | washout_outcome |  |  |
|  | No | 39 | 15.6 | 11 | 12.4 | 28 | 17.4 |  |  |  |  |
|  | Yes | 64 | 25.6 | 17 | 19.1 | 47 | 29.2 |  |  |  |  |
|  | n/a | 147 | 58.8 | 61 | 68.5 | 86 | 53.4 |  |  |  |  |
| Did the authors define when follow-up begins relative to the study entry date? |  |  |  |  |  |  |  |  | followup_begin |  |  |
|  | No | 60 | 24.0 | 20 | 22.5 | 40 | 24.8 |  |  |  |  |
|  | Other | 10 | 4.0 | 7 | 7.9 | 3 | 1.9 |  |  |  |  |
|  | Yes, ON the cohort entry date | 84 | 33.6 | 27 | 30.3 | 57 | 35.4 |  |  |  |  |
|  | Yes, on day X after cohort entry date | 77 | 30.8 | 16 | 18.0 | 61 | 37.9 |  |  |  |  |
|  | n/a | 19 | 7.6 | 19 | 21.3 | - | - |  |  |  |  |
| Did the authors provide criteria used to define the end of follow-up? |  |  |  |  |  |  |  |  | censoring_criteria |  |  |
|  | No | 50 | 20.0 | 27 | 30.3 | 23 | 14.3 |  |  |  |  |
|  | Yes | 176 | 70.4 | 38 | 42.7 | 138 | 85.7 |  |  |  |  |
|  | n/a | 24 | 9.6 | 24 | 27.0 | - | - |  |  |  |  |
| Did the authors clearly define the covariate assessment window relative to the study entry date? |  |  |  |  |  |  |  |  | covariate_assessment_window |  |  |
|  | No | 49 | 19.6 | 18 | 20.2 | 31 | 19.3 |  |  |  |  |
|  | Yes, for all covariates | 156 | 62.4 | 52 | 58.4 | 104 | 64.6 |  |  |  |  |
|  | Yes, for some covariates | 39 | 15.6 | 13 | 14.6 | 26 | 16.1 |  |  |  |  |
|  | n/a | 6 | 2.4 | 6 | 6.7 | - | - |  |  |  |  |
| If a comorbidity/risk score was used, did the authors provide the algorithms for all components of the score? |  |  |  |  |  |  |  |  | comorbidity_score_report |  |  |
|  | No details provided | 22 | 8.8 | 2 | 2.2 | 20 | 12.4 |  |  |  |  |
|  | Provided a citation | 83 | 33.2 | 28 | 31.5 | 55 | 34.2 |  |  |  |  |
|  | Provided some/all details | 15 | 6.0 | 5 | 5.6 | 10 | 6.2 |  |  |  |  |
|  | n/a | 130 | 52.0 | 54 | 60.7 | 76 | 47.2 |  |  |  |  |
|  |  |  |  |  |  |  |  |  |  |  |  |
| **Other study characteristics** |  |  |  |  |  |  |  |  |  |  |  |
| Source of study funding |  |  |  |  |  |  |  |  | Sources_of_funding |  |  |
|  | Govt/Non-profit/Academic | 147 | 58.8 | 56 | 62.9 | 91 | 56.5 |  |  |  |  |
|  | Industry | 74 | 29.6 | 23 | 25.8 | 51 | 31.7 |  |  |  |  |
|  | No funding mentioned | 29 | 11.6 | 10 | 11.2 | 19 | 11.8 |  |  |  |  |
| Type of Journal |  |  |  |  |  |  |  |  | Journal_type |  |  |
|  | Clinical specialty | 151 | 60.4 | 48 | 53.9 | 103 | 64.0 |  |  |  |  |
|  | General clinical | 76 | 30.4 | 35 | 39.3 | 41 | 25.5 |  |  |  |  |
|  | Epidemiology | 16 | 6.4 | 5 | 5.6 | 11 | 6.8 |  |  |  |  |
|  | Other | 7 | 2.8 | 1 | 1.1 | 6 | 3.7 |  |  |  |  |
| Journal Impact Factor (median, interquartile range) |  | 5 | [3, 13] | 5 | [3, 19] | 5 | [3, 9] |  | journal_impact_factor |  |  |
| Author citation index (median, interquartile range) |  | 14 | [6, 29] | 16 | [6, 30] | 13 | [6, 26] |  | first_author_citation_index |  |  |
| Year of publication |  |  |  |  |  |  |  |  | year_of_publication |  |  |
|  | 2011 | 3 | 1.2 | 1 | 1.1 | 2 | 1.2 |  |  |  |  |
|  | 2012 | 13 | 5.2 | 7 | 7.9 | 6 | 3.7 |  |  |  |  |
|  | 2013 | 18 | 7.2 | 10 | 11.2 | 8 | 5.0 |  |  |  |  |
|  | 2014 | 31 | 12.4 | 16 | 18.0 | 15 | 9.3 |  |  |  |  |
|  | 2015 | 33 | 13.2 | 12 | 13.5 | 21 | 13.0 |  |  |  |  |
|  | 2016 | 63 | 25.2 | 26 | 29.2 | 37 | 23.0 |  |  |  |  |
|  | 2017 | 84 | 33.6 | 17 | 19.1 | 67 | 41.6 |  |  |  |  |
|  | 2018 | 5 | 2.0 | 0 | 0.0 | 5 | 3.1 |  |  |  |  |
| Originally conducted by investigators within the same research department as the reproduction team?† |  |  |  |  |  |  |  |  | DoPE |  |  |
|  | No | 239 | 95.6 | 87 | 97.8 | 152 | 94.4 |  |  |  |  |
|  | Yes | 11 | 4.4 | 2 | 2.2 | 9 | 5.6 |  |  |  |  |
| Total number of categories* in which at least one assumption was made |  |  |  |  |  |  |  |  | assumption_total |  |  |
|  | Index date | 93 | 37.2 | 37 | 41.6 | 56 | 34.8 |  |  |  |  |
|  | Inclusion-exclusion criteria | 226 | 90.4 | 73 | 82.0 | 153 | 95.0 |  |  |  |  |
|  | Exposure | 138 | 55.2 | - | - | 138 | 85.7 |  |  |  |  |
|  | Outcome | 146 | 58.4 | 52 | 58.4 | 94 | 58.4 |  |  |  |  |
|  | Follow up | 88 | 35.2 | 37 | 41.6 | 51 | 31.7 |  |  |  |  |
|  | Covariates | 230 | 92.0 | 76 | 85.4 | 154 | 95.7 |  |  |  |  |
|  |  |  |  |  |  |  |  |  |  |  |  |
|  | mean (sd) | 3.7 | (1.2) | 3.1 | (1.2) | 4 | (1.1) |  |  |  |  |
|  | median, IQR | 4 | [3, 4] | 3 | [2, 4] | 4 | [3, 5] |  |  |  |  |
|  |  |  |  |  |  |  |  |  |  |  |  |
| † We ensured that the reproduction team members assigned to the study reproduction were comprised of staff and faculty members not involved with the original publication. | | | | | | | | | | |  |
| * The maximum number of categories was 6 for comparative studies (index date, inclusion-exclusion, exposure, outcome, covariate, follow up).  The maximum number of categories was 5 for descriptive studies. Because descriptive studies did not evaluate a specific exposure, no assumptions were made in that category.  Source data are provided as a Source Data file. | | | | | | | | | | |  |
|  |  |  |  |  |  |  |  |  |  |  |  |

**Supplementary Materials Table 2. Frequency of providing algorithms for operationally defining key study parameters**

|  |  |  |  |  |  |  |  |  |  |  |
| --- | --- | --- | --- | --- | --- | --- | --- | --- | --- | --- |
|  |  |  |  |  |  |  |  |  |  |  |
| **Characteristic** | **Category** | **Inclusion-exclusion** | | **Exposure** | | **Covariate** | | **Outcome** | | **Variable names¹** |
| Did the authors provide diagnosis, procedure, drug codes and/or generic names used to define the parameter? |  | **N =** | **234** | **N =** | **154** | **N =** | **230** | **N=** | **205** | *X*_code_prov |
|  |  | **N** | **%** | **N** | **%** | **N** | **%** | **N** | **%** |  |
|  | Authors provided all codes | 117 | 50.0 | 108 | 70.1 | 67 | 29.1 | 155 | 75.6 |  |
|  | Authors provided some codes | 83 | 35.5 | 29 | 18.8 | 84 | 36.5 | 20 | 9.8 |  |
|  | No codes provided | 34 | 14.5 | 17 | 11.0 | 79 | 34.3 | 30 | 14.6 |  |
|  |  |  |  |  |  |  |  |  |  |  |
| Where were the algorithms to define the criteria provided? |  | **N =** | **242** | **N =** | **144** | **N =** | **194** | **N =** | **210** |  |
|  |  | **N** | **%** | **N** | **%** | **N** | **%** | **N** | **%** |  |
|  | Manuscript | 144 | 59.5 | 108 | 75.0 | 55 | 28.4 | 98 | 46.7 | *X*_codes_manuscript |
|  | Appendix | 86 | 35.5 | 33 | 22.9 | 74 | 38.1 | 79 | 37.6 | *X*_codes_appendix |
|  | Citation | 12 | 5.0 | 3 | 2.1 | 65 | 33.5 | 33 | 15.7 | *X*_codes_citation |
|  |  |  |  |  |  |  |  |  |  |  |
| Did the authors specify whether the code algorithms used to define the criteria were restricted to specific care settings? |  | **N =** | **214** | **N =** | **64** | **N =** | **221** | **N =** | **212** | *X*_pos |
|  |  | **N** | **%** | **N** | **%** | **N** | **%** | **N** | **%** |  |
|  | Not specified | 87 | 40.7 | 39 | 60.9 | 144 | 65.2 | 66 | 31.1 |  |
|  | Specified for at least one | 127 | 59.3 | 25 | 39.1 | 77 | 34.8 | 146 | 68.9 |  |
|  |  |  |  |  |  |  |  |  |  |  |
| Did the authors specify whether codes used to define the criteria were restricted to certain positions? (e.g. primary versus secondary) |  | **N =** | **191** | **N =** | **24** | **N =** | **212** | **N =** | **159** | *X*_codeposition |
|  |  | **N** | **%** | **N** | **%** | **N** | **%** | **N** | **%** |  |
|  | Not specified | 133 | 69.6 | 22 | 91.7 | 199 | 93.9 | 95 | 59.7 |  |
|  | Specified for at least one | 58 | 30.4 | 2 | 8.3 | 13 | 6.1 | 64 | 40.3 |  |
|  |  |  |  |  |  |  |  |  |  |  |
| If non-code definitions (such as age, region, generic name etc.) were used to define the criteria, did the authors clearly define the criteria used? (e.g. how to handle combination generics) |  | **N =** | **215** | **N =** | **98** | **N =** | **236** | **N =** | **86** | *X*_noncodes_prov |
|  |  | **N** | **%** | **N** | **%** | **N** | **%** | **N** | **%** |  |
|  | All information provided | 123 | 57.2 | 39 | 39.8 | 122 | 51.7 | 59 | 68.6 |  |
|  | Some information provided | 77 | 35.8 | 47 | 48.0 | 99 | 41.9 | 20 | 23.3 |  |
|  | Citation provided | 0 | 0.0 | 0 | 0.0 | 2 | 0.8 | 0 | 0.0 |  |
|  | No information provided | 15 | 7.0 | 12 | 12.2 | 13 | 5.5 | 7 | 8.1 |  |
|  |  |  |  |  |  |  |  |  |  |  |
|  |  |  |  |  |  |  |  |  |  |  |
| Note that the N for specific questions changes because we remove the number of studies for which the answer was "not applicable" from the denominator. | | | | | | |  |  |  |  |
| ¹ The italicized X is a tag for the variable name which specifies whether the question refers to inclusion-exclusion, exposure, covariate or outcome.  Source data are provided as a Source Data file. | | | | | |  |  |  |  |  |

**Supplementary Table 3. Variation in magnitude of the absolute difference in coefficient for hazard ratio, risk ratio, or odds ratio between original and reproduction by study characteristics
(relative effect estimate reported in 107 of 118 comparative studies)**

|  | **Number of studies** | **Mean of differences** | **SE** | **P-values** |  |
| --- | --- | --- | --- | --- | --- |
| **Magnitude of original effect estimate** |  |  |  |  |  |
| small (0.8-1.2) | 27 | 0.13 | 0.03 | 0.00 |  |
| medium (0.7-0.8 or 1.2-1.5) | 42 | 0.17 | 0.02 |  |  |
| large (<0.7 or >1.5) | 38 | 0.28 | 0.04 |  |  |
| **Study design diagram provided?** |  |  |  |  |  |
| No | 96 | 0.19 | 0.02 | 0.20 |  |
| Yes | 11 | 0.27 | 0.07 |  |  |
| **Attrition table provided?** |  |  |  |  |  |
| No | 39 | 0.17 | 0.02 | 0.26 |  |
| Yes | 68 | 0.22 | 0.03 |  |  |
| **Did the authors clearly define the study population entry date?** |  |  |  |  |  |
| No | 6 | 0.25 | 0.17 | 0.50 |  |
| Yes | 101 | 0.20 | 0.02 |  |  |
| **Did the authors clearly define the timing of required observable time prior to the study entry date?** |  |  |  |  |  |
| No | 15 | 0.12 | 0.02 | 0.09 |  |
| Yes | 92 | 0.21 | 0.02 |  |  |
| **Did the authors clearly define the timing of the inclusion-exclusion assessment window prior to the study entry date?** |  |  |  |  |  |
| No | 28 | 0.24 | 0.04 | 0.15 |  |
| Yes | 79 | 0.18 | 0.02 |  |  |
| **Did the authors clearly define the timing of the covariate assessment window prior to the study entry date?** |  |  |  |  |  |
| No | 16 | 0.22 | 0.05 | 0.58 |  |
| Yes | 91 | 0.20 | 0.02 |  |  |
| **How many baseline temporal anchors did the authors clearly define prior to the study entry date?** |  |  |  |  |  |
| 0-3 | 48 | 0.23 | 0.03 | 0.11 |  |
| All 4 | 59 | 0.17 | 0.02 |  |  |
| **Did the authors clearly define the washout window for incident exposures relative to the study entry date?** |  |  |  |  |  |
| No | 19 | 0.23 | 0.04 | 0.76 |  |
| n/a | 30 | 0.19 | 0.04 |  |  |
| Yes | 58 | 0.20 | 0.02 |  |  |
| **Did the authors clearly define the washout window for incident outcomes relative to the study entry date?** |  |  |  |  |  |
| No | 18 | 0.20 | 0.04 | 0.06 |  |
| n/a | 57 | 0.16 | 0.03 |  |  |
| Yes | 32 | 0.27 | 0.04 |  |  |
| **Did the authors define when follow-up begins relative to the study entry date?** |  |  |  |  |  |
| No | 29 | 0.18 | 0.03 | 0.49 |  |
| Yes | 78 | 0.21 | 0.02 |  |  |
| **Did the authors provide criteria used to define the end of follow-up?** |  |  |  |  |  |
| No | 10 | 0.12 | 0.03 | 0.19 |  |
| Yes | 97 | 0.21 | 0.02 |  |  |
| **Did the authors address how early refills are handled when defining exposure duration?** |  |  |  |  |  |
| n/a | 50 | 0.20 | 0.03 | 0.26 |  |
| No | 53 | 0.21 | 0.02 |  |  |
| Yes | 4 | 0.05 | 0.02 |  |  |
| **Did the authors address how gaps in days supply between consecutive dispensations or prescriptions are handled when defining exposure duration?** |  |  |  |  |  |
| No | 20 | 0.21 | 0.05 | 0.91 |  |
| n/a | 51 | 0.20 | 0.03 |  |  |
| Yes | 36 | 0.19 | 0.02 |  |  |
| **Did the authors address how extension of hypothesized effect of exposure effect the last days supply was handled when defining exposure duration?** |  |  |  |  |  |
| No | 21 | 0.19 | 0.03 | 0.92 |  |
| n/a | 52 | 0.21 | 0.03 |  |  |
| Yes | 34 | 0.20 | 0.03 |  |  |
| **Were outcome code algorithms provided?** |  |  |  |  |  |
| Authors provided all codes | 73 | 0.20 | 0.02 | 0.72 |  |
| Authors provided some codes | 9 | 0.21 | 0.07 |  |  |
| No codes provided | 13 | 0.16 | 0.04 |  |  |
| n/a | 12 | 0.25 | 0.09 |  |  |
| **Was the outcome place of service specified?** |  |  |  |  |  |
| Not specified | 28 | 0.17 | 0.02 | 0.37 |  |
| Specified for at least one | 68 | 0.20 | 0.03 |  |  |
| n/a | 11 | 0.27 | 0.04 |  |  |
| **Was the outcome diagnosis position specified?** |  |  |  |  |  |
| Not specified | 39 | 0.20 | 0.03 | 0.80 |  |
| Specified for at least one | 44 | 0.19 | 0.03 |  |  |
| n/a | 24 | 0.22 | 0.05 |  |  |
| **Data source** |  |  |  |  |  |
| CPRD | 13 | 0.25 | 0.03 | 0.68 |  |
| MarketScan | 65 | 0.20 | 0.02 |  |  |
| Medicare | 15 | 0.17 | 0.05 |  |  |
| Optum | 14 | 0.18 | 0.05 |  |  |
| **Year of publication** |  |  |  |  |  |
| 2011 | 1 | 0.39 | NA | 0.75 |  |
| 2012 | 2 | 0.07 | 0.07 |  |  |
| 2013 | 1 | 0.30 | NA |  |  |
| 2014 | 4 | 0.25 | 0.05 |  |  |
| 2015 | 15 | 0.15 | 0.05 |  |  |
| 2016 | 28 | 0.18 | 0.03 |  |  |
| 2017 | 51 | 0.21 | 0.03 |  |  |
| 2018 | 5 | 0.26 | 0.12 |  |  |
| **Author citation index (from Scinapse.io)** |  |  |  |  |  |
| <10 | 46 | 0.21 | 0.03 | 0.71 |  |
| 10-19 | 24 | 0.21 | 0.05 |  |  |
| 20+ | 37 | 0.18 | 0.03 |  |  |
| **Journal impact factor** |  |  |  |  |  |
| <3 | 27 | 0.22 | 0.03 | 0.45 |  |
| 4-5 | 44 | 0.21 | 0.03 |  |  |
| 6+ | 32 | 0.16 | 0.03 |  |  |
| Missing | 4 | 0.25 | 0.09 |  |  |
| **Source of funding** |  |  |  |  |  |
| Govt/Non-profit/Academic | 48 | 0.18 | 0.02 | 0.54 |  |
| Industry | 43 | 0.22 | 0.03 |  |  |
| No funding mentioned | 16 | 0.22 | 0.04 |  |  |
| **Author response** |  |  |  |  |  |
| Did not respond | 40 | 0.23 | 0.03 | 0.36 |  |
| Responded with answers | 52 | 0.19 | 0.03 |  |  |
| Responded but declined to answer questions | 4 | 0.24 | 0.10 |  |  |
| E-mail undeliverable | 11 | 0.12 | 0.04 |  |  |
|  |  |  |  |  |  |
| Estimate of effect was hazard ratio, odds ratio, risk ratio, or rate ratio for 107 of 118 comparative studies reproduced.  Source data are provided as a Source Data file. | | | | | |

**Supplementary Table 4. Ten most extreme outliers from reproduction of measures of association**

| **Absolute difference in coefficient** | **Anecdote** |
| --- | --- |
| 1.07 | The reproduction team made assumptions about how the study entry date was defined for the exposed group and the standard of care comparator. They made assumptions about care setting and that the procedure could appear in any position in a claims listing of procedure codes to define exposure. Care setting, code position, and code algorithms were assumed for covariates. The reproduction team made assumptions about how the exposed and comparator cohort were matched. |
| 0.92 | The team made assumptions about how initiation of the exposure and comparator therapies were operationally defined. The algorithms used to define duration of exposure were also assumed. They made assumptions regarding which drugs initiators of therapy were required to be naïve to in order to meet washout criteria. The team assumed that if there were multiple eligible cohort entry dates for a patient, the first eligible cohort entry date in the study period was included. There were numerous assumptions regarding code algorithms, care setting and diagnosis positions used to define inclusion-exclusion criteria and covariates. The diagnosis position for the outcome algorithm was assumed. The team assumed that exposure duration was defined using an algorithm that added 60 days of exposed time after every injection and 30 days added to the days supply for every dispensed oral product. |
| 0.72 | The source data were mapped to the OMOP common data model, but the paper was not clear on version number for the data model. The reproduction team converted the source data to one version of the common data model, but some of the conceptIDs provided by the authors were not mapped in the version the reproduction team used. The team made assumptions about which native codes were included in those mapped conceptIDs. The team made assumptions about algorithms to define duration of exposure given date of dispensing and days supply. The look back window to define some covariates was unclear. The authors referred to the outcome as an incident outcome, but did not specify that they required a washout period during which no outcome was recorded prior to the cohort entry date. The team assumed there was no washout to define an incident outcome and that duration of exposure was based on days supply without adding days to the reported days supply to bridge gaps in exposure or extend exposed days beyond the last dispensation's days supply. The team made assumptions about parameter settings in regularized regression models. Note: This study was registered prior to study implementation, however no protocol was included with registration. |
| 0.66 | The team made assumptions regarding temporality, codes and diagnosis position for inclusion-exclusion criteria and covariates. Note that this data source retroactively updated historical years of data with more older patients and death data. The original cohort was smaller and had wide confidence intervals around the point estimate. The reproduced cohort had tighter confidence intervals and the point estimate was within the bounds of the original 95% CI. |
| 0.56 | The team made assumptions about which exclusion criteria were applied when there was a discrepancy between the criteria reported in the methods text and the attrition table. The assessment window used and algorithms used to define inclusion-exclusion criteria and covariates were assumed. |
| 0.55 | The team made assumptions about which exclusion criteria were applied when there was a discrepancy between the criteria reported in the methods text and the age categories reported in the table describing baseline characteristics. The team made assumptions about the timing of measurement of inclusion-exclusion criteria, including whether there was a minimum baseline window of observable time. Several algorithms used to define inclusion-exclusion criteria were assumed. The team also made assumptions regarding what was considered the index date for cohort entry (time 0), as well as the timing of follow up start and end. |
| 0.53 | The reproduction team made assumptions regarding which generics were included in the class of medications that defined exposure. The code algorithms used to define each generic and algorithms used to define duration of exposure were assumed as well. There were numerous assumptions regarding code algorithms, care setting and diagnosis positions used to define inclusion-exclusion criteria and covariates. The diagnosis position for the outcome algorithm was assumed. The functional form of continuous covariates in the regression model was assumed. The team assumed that exposure duration was defined using an algorithm that added 60 days of exposed time after every injection and 60 days added to the days supply for every dispensed oral product. |
| 0.52 | The timing of exposure ascertainment relative to study entry was unclear, as were the codes used to measure exposure. Inconsistency between lack of age exclusion criterion described in text and age categories to describe the cohort. Lack of clarity in paper resulted in the team making assumptions regarding assessment windows and code algorithms to define inclusion-exclusion criteria and covariates. Assumptions regarding which covariates were included in multivariable model. |
| 0.52 | The team made assumptions regarding the timing and variables used to define a baseline window of observability prior to the index date defining cohort entry. The timing and algorithms used to define covariates were assumed. |
| 0.51 | The team made assumptions about the algorithms used to define exposure, outcome, inclusion-exclusion criteria and covariates |
