# Supplementary material for: Reproducibility of real-world evidence studies using clinical practice data to inform regulatory and coverage decisions
Source: Nat Commun. 2022 Aug 31;13:5126. doi: 10.1038/s41467-022-32310-3 (PMC9430007; doi:10.1038/s41467-022-32310-3)
Supplement: Supplementary file 3 — Description of Additional Supplementary Information [file 41467_2022_32310_MOESM3_ESM.pdf]

**Description of Additional Supplementary Information**

Title: Supplementary Data 1

Description: REPEAT Extraction Form

Title: Supplementary Data 2

Description: Default assumptions and REPEAT standard measures code list

Title: Supplementary Data 3

Description: Author Contact Protocol Data

Title: Supplementary Data 4

Description: Study specific author contact files with details of implementation

Title: Supplementary Data 5

Description: List of reproduced studies

Title: Supplementary Data 6

Description: Analysis file, code, data dictionary
