# Supplementary material for: Reproducibility of real-world evidence studies using clinical practice data to inform regulatory and coverage decisions
Source: Nat Commun. 2022 Aug 31;13:5126. doi: 10.1038/s41467-022-32310-3 (PMC9430007; doi:10.1038/s41467-022-32310-3)
Supplement: Supplementary file 5 — Supplementary Dataset 2 [file 41467_2022_32310_MOESM5_ESM.docx]

***Default assumptions for common parameters that were unclear***

When sufficient details were not provided in the manuscript, we made the following default assumptions:

1. If primary outcome and primary cohort was unclear, we replicated the first reported outcome/result in the abstract.
2. If diagnosis position and care setting was unclear to define outcome, we assumed primary diagnosis position in an inpatient setting.
3. If diagnosis position and care setting was unclear to define exposure/covariates/inclusion and exclusion criteria, we assumed any diagnosis position and any care setting.
   - - 1. MarketScan database: We used Inpatient admission table and Outpatient table.
       2. Optum database: We used Inpatient confinement table and Medical service file.
       3. Medicare database for Rheumatologic studies: We used Medpar file, Outpatient file and Carrier claims file.
       4. Medicare database for NOAC and Diabetes studies: We used Inpatient file, Outpatient file and Carrier claims file unless usage of other files like HHA file, SNF file, and so on is mentioned.
4. If codes are not provided to define variables, then use codes from “standard measure” code set and use only the codes which can be mapped to ontology. The “standard measure” code set can be found in the file xx.
5. If it is unclear whether NDC codes or generic names are used to define drug, we assumed generic name was used.
6. If there was no mention of bridge of coverage gaps in the enrollment, we assumed no gaps.
7. If it was unclear about how combination of drugs was handled, we assumed combination of drugs was used.
8. If it was unclear in the manuscript description, whether exclusion criteria was applied before or after selection of cohort entry date, we assumed the order of exclusion based on flow chart.
9. If it was unclear about grace window and risk window, we assumed 0 days.
10. If it was unclear and manuscript provided either grace window or risk window days, we assumed the same number of days for the other.
11. We started with a library of code algorithms to define patient comorbidities and concomitant medications. ICD-9 Code and generic name based algorithms are listed below. Additional algorithms were identified through literature searches as needed. READ code algorithms for CPRD data were identified from <https://clinicalcodes.rss.mhs.man.ac.uk/> as well as literature searches.

| **Variable** | **Definition** |
| --- | --- |
| 1. Obesity | - ICD-9 diagnosis codes:   278.00, 278.01 539.xx, 649.1x, 649.2x, V85.3x, V85.4x, 43.82, 43.89, 44.31, 44.38, 44.39, 44.68, 44.69, 44.95, 45.51, 45.91  OR   - Prescription claims:   Use of orlistat, lorcaserin, phentermine/topiramate (fixed combo), bupropion/naltrexone (fixed combo), phentermine, diethylpropion, phendimetrazine, benzphetamine, sibutramine, dexfenfluramine, fenfluramine  OR   - CPT/HCPCS codes:   43644, 43645, 43659, 43770, 43842, 43843, 43844, 43845, 43846, 43847, 43999, S2082 |
| 1. Overweight | 278.02, V85.2x |
| 1. Tobacco use | - ICD-9 diagnosis codes:   305.1x, 649.0x, 989.84, V15.82  OR   - CPT/HCPCS codes:   99406, 99407, G0436, G0437, G9016, S9075, S9453, S4995, G9276, G9458, 1034F, 4004F, 4001F  OR   - Prescription claims:   use of varenicline tartrate or nicotine replacement therapy |
| 1. Alcohol abuse or dependence | - ICD-9 diagnosis codes:   291.xx, 303.xx, 305.0x, 571.0x, 571.1x, 571.2x, 571.3x, 357.5x, 425.5x, E860.0x, V11.3x |
| 1. Drug abuse or dependence | - ICD-9 diagnosis codes:   292.xx, 304.xx, 305.2x-305.9x, 648.3x |
| 1. Diabetic retinopathy | - ICD-9 diagnosis codes:   362.0x |
| 1. Diabetes with other ophthalmic manifestations | - ICD-9 diagnosis codes:   250.5x (without mention of 362.01-362.07), 366.41 (diabetic cataract), 365.44 (diabetic glaucoma) |
| 1. Retinal detachment, vitreous hemorrhage, vitrectomy | - ICD-9 diagnosis codes:   361.9x, 379.23  OR   - ICD-9 procedure codes:   14.7x |
| 1. Retinal laser coagulation therapy | - ICD-9 procedure codes:   14.24, 14.34, 14.54   - CPT/HCPCS codes:   67210, 67228 |
| 1. Diabetic neuropathy | - ICD-9 diagnosis codes:   250.6x, 357.2x, 337.1 |
| 1. Diabetic nephropathy | - ICD-9 diagnosis codes:   250.4x, 250.40, 250.41, 250.42, 250.43 |
| 1. Hypoglycemia | - ICD-9 diagnosis codes:   251.1x, 251.20, 962.30  Also, 250.8x as long as none of the following codes are co-occurring diagnoses (i.e. same day): 259.8, 272.7, 681.xx, 682.xx, 686.9x, 707.xx, 709.3, 730.0-730.2, 731.8 |
| 1. **Hyperglycemia** | - ICD-9 diagnosis code:   **790.29** |
| 1. Disorders of fluid electrolyte and acid-base balance | - ICD-9 diagnosis codes:   276.xx |
| 1. Diabetic ketoacidosis | - ICD-9 diagnosis codes:   250.1x |
| 1. Diabetes | - ICD-9 diagnosis codes:   250.x |
| 1. Hyperosmolar hyperglycemic nonketotic syndrome (HONK) | - ICD-9 diagnosis codes:   250.2x |
| 1. Diabetes with peripheral circulatory disorders | - ICD-9 diagnosis codes:   250.7x, 443.81 |
| 1. Diabetic foot | - ICD-9 diagnosis codes:   707.1x |
| 1. Gangrene | - ICD-9 diagnosis codes:   785.4x |
| 1. Lower extremity amputation | - ICD-9 diagnosis codes:   V49.7x (NOT V49.76 or V49.77)   - ICD-9 procedure codes:   84.10-84.17   - CPT/HCPCS codes:   27590, 27591, 27592, 27880, 27881, 27882, 27884, 27886, 27888, 27889, 28800, 28805, 28810, 28820, 28825 |
| 1. Osteomyelitis | - ICD-9 diagnosis codes:   730.xx |
| 1. Skin infections | - ICD-9 diagnosis codes:   680.xx-686.xx |
| 1. Erectile dysfunction | - ICD-9 diagnosis code:   607.84 |
| 1. Diabetes with unspecified complication | - ICD-9 diagnosis codes:   250.9x |
| 1. Diabetes mellitus without mention of complications | - ICD-9 diagnosis codes:   250.0x |
| 1. Gestational diabetes | - ICD-9 diagnosis codes:   648.8x |
| 1. Hypertension (Pre-existing) | - ICD-9 diagnosis codes:   401.x-405.x, 642.0x-642.2x, 642.7x, 642.9x |
| 1. Gestational hypertension | - ICD-9 diagnosis codes:   642.3x |
| 1. Hyperlipidemia | - ICD-9 diagnosis codes:   272.0x-272.4x |
| 1. Ischemic heart disease | - ICD-9 diagnosis codes:   410.xx-414.xx |
| 1. Acute MI | - ICD-9 diagnosis codes:   410.xx |
| 1. ACS/unstable angina | - ICD-9 diagnosis codes:   411.xx |
| 1. Old MI | - ICD-9 diagnosis codes:   412.xx |
| 1. Stable angina | - ICD-9 diagnosis codes:   413.xx |
| 1. Coronary atherosclerosis and other forms of chronic ischemic heart disease | - ICD-9 diagnosis codes:   414.xx |
| 1. Other atherosclerosis | - ICD-9 diagnosis codes:   429.2x (arteriosclerotic cardiovascular disease)  440.9x (generalized and unspecified atherosclerosis) |
| 1. Previous cardiac procedure (CABG or PTCA or Stent) | - ICD9 procedure codes:   00.66, 36.01, 36.02, 36.03, 36.04, 36.05, 36.06, 36.07, 36.09, 36.1x, 36.2x  OR   - CPT/HCPCS codes:   33510 – 33536, 33545, 33572, 92973, 92980, 92981, 92982, 92984, 92995, 92996 |
| 1. History of CABG or PTCA | - ICD-9 diagnosis codes:   V45.81, V45.82 |
| 1. Any stroke | - ICD-9 diagnosis codes:   430.xx, 431.xx, 433.xx, 434.xx, 436.xx |
| 1. Ischemic stroke (w and w/o mention of cerebral infarction) | - ICD-9 diagnosis codes:   433.xx, 434.xx, 436.xx |
| 1. Hemorrhagic stroke | - ICD-9 diagnosis codes:   430.xx, 431.xx |
| 1. TIA | - ICD-9 diagnosis codes:   435.xx |
| 1. Other cerebrovascular disease | - ICD-9 diagnosis codes:   432.xx, 437.xx |
| 1. Late effects of cerebrovascular disease | - ICD-9 diagnosis codes:   438.xx |
| 1. Cerebrovascular procedure | *Carotid bypass -*   - ICD9 procedure codes: 39.28   *Cerebrovascular revascularization -*   - ICD9 procedure codes:   00.61 - 00.65, 38.11, 38.12  OR   - CPT:   35301, 35390, 35501, 35601, 35901, 0075T, 0076T, 37215, 37216 |
| 1. Heart failure (CHF) | - ICD-9 diagnosis codes:   428.x, 398.91, 402.01, 402.11, 402.91, 404.01, 404.11, 404.91, 404.03, 404.13, 404.93 |
| 1. Peripheral Vascular disease or PVD surgery | *Peripheral vascular disease -*   - ICD9 diagnosis codes:   440.20 – 440.24, 440.29 – 440.32, 440.3, 440.4, 443.9  OR  *Lower-extremity endarterectomy, stenting, angioplasty, or atherectomy -*   - ICD9 procedure codes:   38.18, 38.19  OR   - CPT/HCPCS codes:   35454, 35456, 35459, 35470, 35473, 35474, 35482, 35483, 35485, 35492, 35493, 35495, 37207, 37208, 37220-27235  OR   - Lower-extremity bypass -   ICD9 procedure codes:  39.25, 39.29   - CPT/HCPCS codes:   35351, 35355, 35361, 35363, 35371, 35372, 35521, 35533, 35541, 35546, 35548, 35549, 35551, 35556, 35558, 35563, 35565, 35566, 35571, 35621, 35623, 35641, 35646, 35647, 35651, 35654, 35656, 35661, 35663, 35666, 35671, 35570, 35582, 35583, 35585, 35587, 35637, 35638, 35681, 35682, 35683  OR  *Other peripheral vascular surgery -*   - ICD9 procedure codes:   38.08, 38.09, 38.38, 38.39,38.48, 38.49, 39.5x, 39.9x   - CPT/HCPCS codes:   35256, 35286, 35381, 35879 |
| 1. Atrial fibrillation | - ICD-9 diagnosis codes:   427.3x |
| 1. Other cardiac dysrhythmia | - ICD-9 diagnosis codes:   427.xx, exclude 427.5x (cardiac arrest) and 427.3x |
| 1. Cardiac conduction disorders | - ICD-9 diagnosis codes:   426.xx |
| 1. Other CVD | - ICD-9 diagnosis codes:   390.xx -398.xx, 420.xx -425.xx, 441.xx -447.xx (except 442.1x, 443.81, 443.9x) |
| 1. Edema | - ICD-9 diagnosis codes:   782.3x |
| 1. COPD | - ICD-9 diagnosis codes:   491.xx, 492.xx, or 496.xx |
| 1. Asthma | - ICD-9 diagnosis codes:   493.xx |
| 1. Obstructive sleep apnea | - ICD-9 diagnosis codes:   327.23 |
| 1. Pneumonia | - ICD-9 diagnosis codes:   480.xx – 486.xx, 487.0x, 507.xx |
| 1. *Acute Renal Disease* | - ICD-9 diagnosis codes:   572.4x, 580.xx, 584.xx, 580.0x, 580.4x, 580.89, 580.9x, 582.4x, 642.1x, 791.2x, 791.3x |
| 1. *Chronic Renal Insufficiency* | - ICD-9 diagnosis codes:   582.xx, 583.xx, 585.xx, 586.xx, 587.xx, 642.1x |
| 1. Chronic kidney disease | - ICD-9 diagnosis codes:   585.xx |
| 1. CKD Stage 3-4 | - ICD-9 diagnosis codes:   585.3x-585.4x |
| 1. *Hypertensive Nephropathy* | - ICD-9 diagnosis codes:   403.xx, 404.xx |
| 1. *Miscellaneous Renal Insufficiency* | - ICD-9 diagnosis codes:   274.10, 440.1x, 442.1x, 453.3x, 581.xx, 593.xx, 753.0x, 753.3x, 866.00, 866.01, 866.1x |
| 1. Liver disease | - ICD-9 diagnosis codes:   070.xx, 570.xx- 573.xx, 456.0x-456.2x, 576.8x, 782.4x, 789.5x  OR   - ICD-9 procedure codes:   39.1x, 42.91 |
| 1. Irritable bowel syndrome | - ICD-9 diagnosis codes:   564.1x |
| 1. Crohn’s disease | - ICD-9 diagnosis codes:   555.xx |
| 1. Ulcerative colitis | - ICD-9 diagnosis codes:   556.xx |
| 1. Osteoarthritis | - ICD-9 diagnosis codes:   715.xx |
| 1. Rheumatoid arthritis | - ICD-9 diagnosis codes:   714.0x, 714.1x, 714.2x |
| 1. Other arthritis, arthropathies and musculoskeletal pain | - ICD-9 diagnosis codes:   710.xx−714.xx, 716.xx−719.xx, 725.xx−729.xx (excluding 729.2x, 714.0x, 714.1x, 714.2x)] |
| 1. Dorsopathies | - ICD-9 diagnosis codes:   720.xx-724.xx |
| 1. Back and neck pain | - ICD-9 diagnosis codes:   720.0x, 720.1x, 720.2x, 721.3x, 722.10, 722.32, 722.5x, 722.83, 722.93, 724.00, 724.02, 724.2x, 724.5x, 724.6x, 724.70, 724.71, 724.79, 720.81, 720.89, 720.9x, 721.0x, 721.2x, 721.5x, 721.6x, 721.7x, 721.8x, 721.90, 722.11, 722.30, 722.31, 722.39, 722.4x, 722.6x, 722.80, 722.81, 722.82, 722.90, 722.91, 722.92, 723.xx (except 723.4x), 724.01, 724.1x, 724.8x, 724.9x |
| 1. Fibromyalgia | - ICD-9 diagnosis codes:   729.1x |
| 1. Fractures | - ICD-9 diagnosis codes:   733.1x, 800.xx-829.xx |
| 1. Falls | - ICD-9 diagnosis codes:   E880.0x-E888.9x (accidental falls)  V15.88 (history of fall) |
| 1. Osteoporosis | - ICD-9 diagnosis codes:   733.0x  OR   - Prescription claims:   use of bisphosphonates (see below for definition), calcitonin, raloxifene, or teriparatide |
| 1. Hyperthyroidism | - ICD-9 diagnosis codes:   242.1x, 242.3x, 242.9x |
| 1. Hypothyroidism | - ICD-9 diagnosis codes:   243.xx, 244.xx |
| 1. Other disorders of thyroid gland | - ICD-9 diagnosis codes:   240.xx – 246.xx, excluding hyperthyroidism and hypothyroidism |
| 1. Essential tremor | - ICD-9 diagnosis codes:   333.1x |
| 1. Neuropathic Pain | - ICD-9 diagnosis codes:   053.1x, 337.1x, 337.2x, 250.6x, 357.2, 350.1x, 350.2x, 352.1x, 353.xx, 354.xx, 355.xx, 357.xx, 729.2x, 721.1x, 721.41, 721.42, 721.91, 722.7x, 723.4x, 724.3x, 724.4 x |
| 1. Depression | - ICD-9 diagnosis codes:   293.83, 296.2x. 296.3x, 298.0x, 300.4x, 309.0x, 309.1x, 309.28, 311.xx |
| 1. Anxiety | - ICD-9 diagnosis codes:   293.84, 300.0x, 300.2x, 300.3x, 309.24, 308.0x, 309.81 |
| 1. Sleep_Disorder | - ICD-9 diagnosis codes:   307.4x, 327.0x, 327.2x 780.5x, 347.xx |
| 1. Dementia | - ICD-9 diagnosis codes:   290.xx, 294.xx, 330.xx, 331.xx |
| 1. Delirium | - ICD-9 diagnosis codes:   290.11, 290.3x, 290.41, 291.0x, 292.81, 293.xx, 348.3x, 349.82 |
| 1. Psychosis | - ICD-9 diagnosis codes:   290.8x, 290.9x, 295.xx, 297.xx, 298.xx, 299.xx, 780.1x |
| 1. Bipolar disorder | - ICD-9 diagnosis codes:   296.0x, 296.1x, 296.4x, 296.5x, 296.6x, 296.7x, 296.8x, 296.99 |
| 1. Schizophrenia | - ICD-9 diagnosis codes:   295.xx |
| 1. Personality disorder | - ICD-9 diagnosis codes:   301.xx |
| 1. Adjustment disorder | - ICD-9 diagnosis codes:   309.21-309.23, 309.29, 309.3x, 309.4x, 309.82, 309.83, 309.89, 309.9x |
| 1. Anxiety | - ICD-9 diagnosis codes:   293.84, 300.0x, 300.2x, 300.3x, 309.24, 308.0x, 309.81, 313.0x, 300.02 |
| 1. ADHD | - ICD-9 diagnosis codes:   312.xx, 314.xx |
| 1. Frailty | - ICD-9 diagnosis codes:   781.2, 783.2, 783.7, V15.88, 780.7, 728.2, 728.87, 707.0, 707.2  OR   - CPT/HCPCS codes:   E0100, E0105, E0130, E0135, E0140, E0141, E0143, E0144, E0147-E0149, E0160-E0171 OR T1000-T1005, T1019-T1022, T1030, T1031  OR   - Additional HCPCS codes to include:   *Wheelchairs, components, and accessories -*  K0001-K0462, K0669  *Transportation services including ambulance -*  A0021-A0999  *Hospital beds and associated supplies -*  E0250-E0373  *Accessories for oxygen delivery devices -*  E1353-E1406 |
| 1. Non-frailty | - CPT/HCPCS codes:   *Screening examinations and disease management training -*  G0101-G0124  *Vaccine administration -*  G0008-G0010  *Miscellaneous drugs and tests -*  Q0035-Q0144  *Preventive medicine services (E&M) -*  99381-99429  *Ophthalmology (medicine) -*  92002-92499  *Chiropractic manipulative treatment (medicine) -*  98940-98943  *Vaccines, toxoids (medicine) -*  90476-90749  *Male genital system (surgery) -*  54000-55899  *Lower abdomen (anesthesia) -*  00800-00882  OR   - ICD-9 diagnosis codes:   *Persons without reported diagnosis encountered during examination and investigation of individuals and populations -*  V70-V82  *Diseases of male genital organs -*  600-608  *Benign neoplasms -*  210–229  *Neoplasms of uncertain behavior -*  235–238  *Disorders of the eye and adnexa -*  360-379  *Persons with potential health hazards related to communicable diseases*  V01-V09 |
| 1. ACE inhibitor | - Prescription claims:   Benazepril, captopril, enalapril, fosinopril, lisinopril, moexipril, perindopril, quinapril, ramipril, trandolapril |
| 1. ARB | - Prescription claims:   Azilsartan, candesartan, eprosartan, irbesartan, losartan, olmesartan, telmisartan, valsartan |
| 1. Beta blocker | - Prescription claims:   Acebutolol, atenolol, betaxolol, bisoprolol, carteolol, carvedilol, esmolol, labetalol, metoprolol tartrate, metoprolol succinate, propranolol, penbutolol, pindolol, nadolol, nebivolol, sotalol, timolol |
| 1. Calcium channel blocker | - Prescription claims:   Diltiazem, mibefradil, verapamil, amlodipine, clevidipine, bepridil, felodipine, isradipine, nicardipine, nifedipine, nimodipine, nisoldipine |
| 1. Thiazides | - Prescription claims:   Benzthiazide, chlorothiazide, chlorthalidone, cyclothiazide, hydrochlorothiazide, hydroflumethiazide, indapamide, methyclothiazide, metolazone, polythiazide, quinethazone, trichlormethiazide, bendroflumethiazide |
| 1. Loop diuretics | - Prescription claims:   Furosemide, bumetanide, torsemide, ethacrynic acid |
| 1. Other diuretics | - Prescription claims:   Amiloride, eplerenone, spironolactone, triamterene |
| 1. Nitrates | - Prescription claims:   Nitroglycerin, isosorbide dinitrate, isosorbide mononitrate, ranolazine |
| 1. Other hypertension drugs | - Prescription claims:   Doxazosin, eplerenone, prazosin, terazosin, clonidine, guanabenz, guanadrel, guanethidine, guanfacine, hydralazine, methyldopa, metyrosine, reserpine, minoxidil, aliskiren |
| 1. Digoxin | - Prescription claim:   Digoxin |
| 1. Antiarrhythmic drugs | - Prescription claims:   Amiodarone, dronedarone, flecainide, ibutilide, procainamide, propafenone, quinidine disopyramide, dofetilide, mexiletine, moricizine,  tocainide |
| 1. COPD or asthma medications | - Prescription claims:   Fluticasone/salmeterol (fixed combo), budesonide/formoterol (fixed combo), mometasone/formoterol (fixed combo), aformoterol, formoterol, salmeterol, albuterol, levalbuterol, metaproterenol, pirbuterol, terbutaline, ipratropium, tiotropium, theophylline, montelukast, zafirlukast, zileuton, aclidinium, indacaterol, olodaterol, umeclidinium |
| 1. Statin | - Prescription claims:   Atorvastatin, fluvastatin, lovastatin, pravastatin, simvastatin, rosuvastatin, lovastatin–niacin, ezetimibe–simvastatin, pravastatin- aspirin, pitavastatin |
| 1. Other lipid-lowering drugs | - Prescription claims:   Niacin, nicotinic acid, niacinamide, fenofibrate, gemfibrozil, cholestyramine, colesevelam, colestipol, ezetimibe alone |
| 1. Antiplatelet | - Prescription claims:   Aspirin alone, clopidogrel, prasugrel, ticlopidine, aspirin–dipyridamole, dipyridamole alone, cilostazol, ticagrelor |
| 1. Anticoagulants | Prescription claims:  Warfarin, dabigatran, rivaroxaban, apixaban |
| 1. Heparin and other low-molecular weight heparins | - Prescription claims:   Heparin, dalteparin, enoxaparin, tinzaparin |
| 1. NSAIDs | - Prescription claims:   Diclofenac, etodolac, flurbiprofen, ketorolac, ibuprofen, indomethacin, meloxicam, naproxen, piroxicam, sulindac, Celecoxib, rofecoxib, valdecoxib |
| 1. Oral corticosteroids | - Prescription claims:   Cortisone, hydrocortisone, prednisone, prednisolone, methylprednisolone, triamcinolone, dexamethasone, betamethasone |
| 1. Bisphosphonates | - Prescription claims:   Alendronate, risedronate, ibandronate or etidronate, zoledronic acid, pamidronic acid, pamidronate |
| 1. Opioids | - Prescription claims:   Butorphanol, codeine, fentanyl, hydrocodone, hydromorphone, levorphanol, methadone, meperidine, morphine, oxycodone, oxymorphone, pentazocine, propoxyphene, tapentadol, tramadol |
| 1. Antidepressants | - Prescription claims:   Paroxetine, Citalopram, Escitalopram, Fluoxetine, Fluvoxamine, Sertraline, Venlafaxine, Desvenlafaxine, Levomilnacipran, Duloxetine, Amitriptyline, Amoxapine, Clomipramine, Desipramine, Doxepin, Imipramine, Maprotiline, Nortriptyline, Protriptyline, Trimipramine, Bupropion, Mirtazapine, Nefazodone, Trazodone, Phenelzine, Isocarboxazid, Tranylcypromine, Vilazodone, Vortioxetine |
| 1. Antipsychotics | - Prescription claims:   Clozapine, risperidone, olanzapine, quetiapine, aripiprazole, ziprasidone, acetophenazine, chlorpromazine, fluphenazine, mesoridazine, perphenazine, promazine, thioridazine, trifluoperazine, triflupromazine, chlorprothixene, haloperidol, loxapine, molindone, pimozide, thiothixene, asenapine, iloperidone, lurasidone, paliperidone, propiomazine, triflupromazine, brexpiprazole, cariprazine, pimavanserin |
| 1. Anticonvulsants | - Prescription claims:   Carbamazepine, divalproex, eslicarbazepine, ethotoin, ethosuximide, ezogabine, felbamate, fosphenytoin, gabapentin, lacosamide, lamotrigine, levetiracetam, mephenytoin, mephobarbital, methsuximide, oxcarbazepine, perampanel, phenobarbital, phenytoin, pregabalin, primidone, rufinamide, tiagabine, topiramate, valproic acid, vigabatrin, zonisamide |
| 1. Lithium | - Prescription claims:   Lithium carbonate, lithium citrate |
| 1. Benzodiazepines | - Prescription claims:   Alprazolam, chlordiazepoxide, clonazepam, clorazepate, diazepam, estazolam, flurazepam, halazepam, lorazepam, midazolam, oxazepam, prazepam, quazepam, temazepam, triazolam |
| 1. Other anxiolytics/hypnotics | - Prescription claims:   Eszopiclone, zaleplon, zolpidem, chloral hydrate, diphenhydramine, doxylamine, ethclorvynol, glutethimide, methaqualone, buspirone. Hydroxyzine, meprobamate, chloral hydrate, ramelteon, suvorexant |
| 1. Agents for dementia | - Prescription claims:   Ergoloid, donepezil, rivastigmine, galantamine, memantine, tacrine |
| 1. Antiparkinson agents | - Prescription claims:   Benztropine, biperiden, procyclidine, trihexyphenidyl, tolcapone, amantadine, levodopa, pergolide, pramixepole, ropinirole, rotigotine, carbidopa/levodopa, rasagiline, selegiline, carbidopa |
| 1. Barbiturates | - Prescription claims:   Amobarbital sodium or amobarbital, Butabarbital sodium or butabarbital, Pentobarbital sodium or pentobarbital, butalbital, Secobarbital, buspirone, meprobamate |
| 1. Non-insulin antidiabetic medications | - Prescription claims:   Acarbose, acetohexamide, albiglutide, alogliptin, canagliflozin, chlorpropamide, dapagliflozin, dulaglutide, empagliflozin, exenatide, glimepiride, glipizide, glyburide, linagliptin, liraglutide, lixisenatide, metformin, miglitol, nateglinide, pioglitazone, pramlintide, repaglinide, rosiglitazone, saxagliptin, sitagliptin, tolazamide, tolbutamide, troglitazone |
| 1. Endocrinologist visit | - Provide codes:   *United: Use PROVCAT_CD variable from Medical service file*  ENDOCRINOLOGIST -  0459, 0460, 0461, 0462, 1143, 1747, 1824, 1959, 2365, 2597, 2800, 2860, 3238, 4160  PEDIATRIC ENDOCRINOLOGIST -  0466, 0467, 0468, 0469, 1251, 2310, 3042, 3222, 3265, 4005, 4016  ENDOCRINOLOGIST (DIABETES SPECIALIST) -  0458, 0463, 0464, 0465, 1429, 1923, 2055, 2704, 3077, 3589  *MarketScan: Use STDPROV variable from both Inpatient and Outpatient file*  270, 433  *Medicare: Line HCFA Provider Specialty Code from Carrier claims file*  46 |
| 1. Internal medicine/family medicine visits | - Provider codes:   *United: Use PROVCAT_CD variable from Medical service file*  0308, 0311, 0312, 0313, 0314, 0316, 0317, 0318, 0319, 0326, 0327, 0329, 0330, 0331, 0332, 0333, 0334, 0335, 0336, 0337, 0338, 0339, 0340, 0341, 0342, 1046, 1113, 1127, 1133, 1419, 1423, 1643, 1646, 1661, 1673, 1694, 1697, 1719, 1721, 1726, 1743, 1755, 1764, 1785, 1790, 1791, 1832, 1854, 1860, 1864, 1865, 1903, 1927, 1940, 2050, 2068, 2073, 2189, 2208, 2219, 2228, 2233, 2249, 2254, 2280, 2286, 2313, 2314, 2324, 2340, 2354, 2359, 2373, 2375, 2378, 2384, 2392, 2403, 2446, 2485, 2574, 2607, 2610, 2613, 2614, 2615, 2628, 2644, 2677, 2736, 2790, 2811, 2826, 2835, 2837, 2842, 2889, 2904, 2930, 2978, 2996, 3122, 3150, 3241, 3268, 3350, 3531, 3564, 3765, 3777, 3812, 4135  *MarketScan: Use STDPROV variable from both Inpatient and Outpatient file*  204, 240, 825, 845  *Medicare: Line HCFA Provider Specialty Code from Carrier claims file*  46 |
| 1. Cardiologist visit | - Provider codes:   *United: Use PROVCAT_CD variable from Medical service file*  0400, 0401, 0402, 0403, 0404, 0405, 0407, 0408, 0409, 0410, 0411, 0412, 0416, 0418, 0419, 0420, 0421, 1107, 1108, 1123, 1135, 1162, 1212, 1253, 1473, 1582, 1629, 1641, 1728, 1757, 1856, 1947, 2082, 2115, 2559, 2653, 2804, 2810, 2854, 2891, 2983, 3073, 3137, 3249, 3259, 3267, 3534, 3780, 3800, 3818, 3848, 3854, 4020, 4120  *MarketScan: Use STDPROV variable from both Inpatient and Outpatient file*  250, 440  *Medicare: Line HCFA Provider Specialty Code from Carrier claims file*  06 |
| 1. Electrocardiogram | - ICD-9 procedure codes:   89.51, 89.52  OR   - CPT/HCPCS codes:   93000, 93005, 93010 |
| 1. Use of glucose test strips | - CPT/HCPCS code:   82948 |
| 1. HbA1c tests ordered | - CPT/HCPCS code:   83036 |
| 1. Glucose tests ordered | - CPT/HCPCS codes:   82947, 82948, 82962 |
| 1. Lipid tests ordered | - CPT/HCPCS codes:   *LDL (direct and calculated) -*  83721, 83701, 83704, 80061  *HDL -*  83718, 80061  *Total Cholesterol -*  82465, 80061  *Triglycerides -*  84478, 80061 |
| 1. Creatinine/BUN tests ordered | - CPT/HCPCS code:   84520 |
| 1. Number of tests for microalbuminuria | - CPT/HCPCS codes:   82043, 82570 |
