# Supplementary material for: Reproducibility of real-world evidence studies using clinical practice data to inform regulatory and coverage decisions
Source: Nat Commun. 2022 Aug 31;13:5126. doi: 10.1038/s41467-022-32310-3 (PMC9430007; doi:10.1038/s41467-022-32310-3)
Supplement: Supplementary file 6 — Supplementary Dataset 3 [file 41467_2022_32310_MOESM6_ESM.docx]

***Author contact protocol***

Guidelines for contacting corresponding authors:

1. Log all dates of attempted contact in tracking file
2. Send no more than 3 e-mails, spaced 1 week apart, to elicit a response from the corresponding author.
3. With each e-mail, include:
4. Excel file containing replication assumptions, cohort creation and analysis summary protocol, Table 1 of baseline characteristics and outcome results for replication and original study, code algorithms used to create measures for exposure, inclusion-exclusion criteria, covariates, and outcomes.
5. Unredacted PDF of the original study.
6. If there is an away message, wait until the day after the person is scheduled to return before sending the next e-mail.
7. If the corresponding author:
   1. does not respond after 3 attempts, record “non-responder” in tracking file.
   2. declines to provide more information, do not send more e-mails, record the date and record “decline” in tracking file.
   3. responds positively, connect the author with the relevant REPEAT team members via e-mail or schedule a call as appropriate.
      1. Record all email correspondences and summaries of each phone call with authors in the tracking file.
