# Supplementary material for: Reproducibility of real-world evidence studies using clinical practice data to inform regulatory and coverage decisions
Source: Nat Commun. 2022 Aug 31;13:5126. doi: 10.1038/s41467-022-32310-3 (PMC9430007; doi:10.1038/s41467-022-32310-3)
Supplement: Supplementary file 9 — Supplementary Dataset 6 [file 41467_2022_32310_MOESM9_ESM.zip › Supplementary Data 6 Analysis file, code, data dictionary/Demo and Data Dictionary.docx]

**Demo and Data Dictionary**

**Instructions for use:**

1. Open RMarkdown file
2. Change working directory filepath to the filepath where the provided datasets are stored
3. Run RMarkdown file (expected run time <5 min)
4. Expected output – plots for manuscript

Note: The provided data is not identical to the data used to produce tables and figures for the manuscript. It has been redacted to remove small cell sizes, in accordance with the study team’s data use agreements.

| **Data dictionary:** |  |
| --- | --- |
|  |  |
| **Baseline cell suppressed.csv** | This dataset is different from the analytic dataset used to produce results for the paper. Cell sizes indicative of patients counts <11 have been redacted for Medicare data. Note that sample sizes for Medicare data may be non-integer. When extracting data from Medicare, we used criteria that included everyone based on health condition rather than selection based on a random sample indicator. If the authors reported using a random 5 or 20% sample, we applied that fraction to our sample size counts in the comparison of sample sizes between the original and reproduction. |
| **Variable Name** | **Variable Description** |
| studyid | Study identifier |
| factor.studyid | Study identifier as a factor variable |
| exposure | Descriptive name of exposure |
| matched | Indicator of whether the row reflects a matched population or not |
| original_variable_name | Original name of variable in the published paper |
| variable_type | Type of variable (continuous, categorical, binary, etc.) |
| variable_level | Labels for categorical variable categories |
| exposure_of_interest | Indicator of whether the row reflects descriptive statistics for the exposure of interest |
| original_percent_mean | The percent or mean reported in the baseline descriptive table for the published paper |
| original_sd | The standard deviation reported in the baseline descriptive table for the published paper |
| reproduction_variable_label | Name of variable used in the reproduction |
| reproduction_percent_mean | The percent or mean reported in the baseline descriptive table for the reproduction |
| reproduction_sd | The standard deviation reported in the baseline descriptive table for the reproduction |
| codes_provided | Indicator of whether the covariate algorithm codes were provided |
| absdiff | Difference in baseline characteristic percent or mean (original - reproduction) |
| pooled_std | Pooled standard deviation |
| stddiff | Standardized difference between baseline characteristic percent or mean (original - reproduction) |
| datasource | Data Source |
| source_data_range | Were the years of source data available for the study specified? |
| sourcedata_cdm | Was source data converted to a common data model? |
| design_diagram | Was a design diagram provided? |
| attrition_table | Was an attrition table provided? |
| missing_data_method | Did the authors specify how missing data or out of range values were handled? |
| index_date | Did the authors clearly define the study entry date? |
| number_entries | Did the authors specify how many times an individual could enter the study population? |
| enrollment_window | Did the authors clearly define the timing of required observable time prior to the study entry date? |
| enrollment_coverage_rc | Did the authors report whether (and how) coverage gaps in observable time were bridged? |
| inclusion_exclusion_window | Did the authors clearly define the time window during which inclusion-exclusion criteria are assessed relative to the study entry date? |
| order_exclusions_re_index | Did the authors specify when exclusion criteria are applied relative to selection of the study entry date? |
| exposure_type | Did the authors specify the type of exposure being measured? |
| washout_exposure | Did the authors clearly define the washout window for incident exposures relative to the study entry date? |
| early_refills | Did the authors address how early refills are handled when defining exposure duration? |
| exposure_gap | Did the authors address how gaps in days supply between consecutive dispensations or prescriptions are handled when defining exposure duration? |
| exposure_extension | Did the authors address how extension of hypothesized effect of exposure effect the last days supply was handled when defining exposure duration? |
| washout_outcome | Did the authors clearly define the washout window for incident outcomes relative to the study entry date? |
| followup_begin | Did the authors define when follow-up begins relative to the study entry date? |
| censoring_criteria | Did the authors provide the censoring criteria used to define the end of follow-up? |
| covariate_assessment_window | Did the authors clearly define the covariate assessment window relative to the study entry date? |
| comorbidity_score_report | If a comorbidity/risk score was used, did the authors provide the algorithms for all components of the score? |
| Sources_of_funding | Source of study funding |
| Journal_type | Type of Journal |
| journal_impact_factor | Journal Impact Factor |
| first_author_citation_index | Author citation index |
| year_of_publication | Year of publication |
| Author.response | Responsiveness of author to reproduction team queries about assumptions made during reproduction |
| DoPE | Originally conducted by investigators within the same research group as the replication team? |
| X_code_prov | Did the authors provide diagnosis, procedure, drug codes and/or generic names used to define the parameter? |
| X_codes_manuscript | Algorithms to define the parameter were provided in the manuscript |
| X_codes_appendix | Algorithms to define the parameter were provided in the appendix |
| X_codes_citation | Algorithms to define the parameter were provided in a citation |
| X_pos | Did the authors specify whether the code algorithms used to define inclusion-exclusion criteria were restricted to specific care settings? |
| X_code_position | Did the authors specify whether a specific code position was used to define the parameter? (e.g. primary position vs secondary) |
| X_non_codes_prov | Did the author provide details of non-clinical code based algorithms used to define parameter? |
|  |  |
| **Outcomes.csv** |  |
| studyid | Study identifier |
| original_outcome_name | Description of the outcome variable |
| model | Description of the outcome model |
| original_pt_est | Published point estimate from the original paper |
| original_lower_95 | Published lower 95% confidence interval bound from the original paper |
| original_upper_95 | Published upper 95% confidence interval bound from the original paper |
| original_p_value | P-value from the original paper |
| reproduction_pt_est | Reproduction point estimate |
| reproduction_lower_95 | Reproduction lower 95% confidence interval bound |
| reproduction_upper_95 | Reproduction upper 95% confidence interval bound |
| reproduction_p_value | Reproduction p-value |
| measure_type | Type of outcome measure |
| original_stdev | Standard deviation from original publication |
| reproduction_stdev | Standard deviation from reproduction |
| absdiff | Difference between original and reproduction statistic (difference in log coefficient for relative effect measures) |
| pooled_std | Pooled standard deviation |
| stddiff | Standardized difference |
| adjusted | Indicator of whether statistic was adjusted for confounding |
| smaller_pt_est | Indicator of whether the reproduction estimate of effect was smaller than the original |
| sameside | Indicator of whether the reproduction estimate was on the same side of null as the original |
| original_null | Indicator of whether the original effect estimate was null |
| reproduction_null | Indicator of whether the reproduction effect estimate was null |
| samesidesig | Indicator of whether the original effect estimate and reproduction estimate were on the same side of null and had the same statistical significance (0.05 threshold) |
| original_p | P-value for original effect estimate |
| reproduction_p | P-value for reproduction effect estimate |
| overlap | Indicator of whether there is overlap in confidence intervals between original and reproduction effect estimates |
| primary | Indicator of whether the result is the primary effect estimate reproduced |
| absdiff10 | Indicator of difference in coefficient greater thatn 0.1 (original - reproduction) |
| absdiff20 | Indicator of difference in coefficient greater thatn 0.2 (original - reproduction) |
| absabsdiff | \| log(coef original) - log(coef reproduction) \| |
| factor.studyid | Study identifier as a factor variable |
| matched | Indicator of whether the statistic comes from a matched data set |
| datasource | Data Source |
| source_data_range | Were the years of source data available for the study specified? |
| sourcedata_cdm | Was source data converted to a common data model? |
| design_diagram | Was a design diagram provided? |
| attrition_table | Was an attrition table provided? |
| missing_data_method | Did the authors specify how missing data or out of range values were handled? |
| index_date | Did the authors clearly define the study entry date? |
| number_entries | Did the authors specify how many times an individual could enter the study population? |
| enrollment_window | Did the authors clearly define the timing of required observable time prior to the study entry date? |
| enrollment_coverage_rc | Did the authors report whether (and how) coverage gaps in observable time were bridged? |
| inclusion_exclusion_window | Did the authors clearly define the time window during which inclusion-exclusion criteria are assessed relative to the study entry date? |
| order_exclusions_re_index | Did the authors specify when exclusion criteria are applied relative to selection of the study entry date? |
| exposure_type | Did the authors specify the type of exposure being measured? |
| washout_exposure | Did the authors clearly define the washout window for incident exposures relative to the study entry date? |
| early_refills | Did the authors address how early refills are handled when defining exposure duration? |
| exposure_gap | Did the authors address how gaps in days supply between consecutive dispensations or prescriptions are handled when defining exposure duration? |
| exposure_extension | Did the authors address how extension of hypothesized effect of exposure effect the last days supply was handled when defining exposure duration? |
| washout_outcome | Did the authors clearly define the washout window for incident outcomes relative to the study entry date? |
| followup_begin | Did the authors define when follow-up begins relative to the study entry date? |
| censoring_criteria | Did the authors provide the censoring criteria used to define the end of follow-up? |
| covariate_assessment_window | Did the authors clearly define the covariate assessment window relative to the study entry date? |
| comorbidity_score_report | If a comorbidity/risk score was used, did the authors provide the algorithms for all components of the score? |
| Sources_of_funding | Source of study funding |
| Journal_type | Type of Journal |
| journal_impact_factor | Journal Impact Factor |
| first_author_citation_index | Author citation index |
| year_of_publication | Year of publication |
| Author.response | Responsiveness of author to reproduction team queries about assumptions made during reproduction |
| DoPE | Originally conducted by investigators within the same research group as the replication team? |
| X_code_prov | Did the authors provide diagnosis, procedure, drug codes and/or generic names used to define the parameter? |
| X_codes_manuscript | Algorithms to define the parameter were provided in the manuscript |
| X_codes_appendix | Algorithms to define the parameter were provided in the appendix |
| X_codes_citation | Algorithms to define the parameter were provided in a citation |
| X_pos | Did the authors specify whether the code algorithms used to define inclusion-exclusion criteria were restricted to specific care settings? |
| X_code_position | Did the authors specify whether a specific code position was used to define the parameter? (e.g. primary position vs secondary) |
| X_non_codes_prov | Did the author provide details of non-clinical code based algorithms used to define parameter? |
|  |  |
| **Sample size.csv** | Note that sample sizes for Medicare data may be non-integer. When extracting data from Medicare, we used criteria based on health condition rather than a random sample. If the authors reported using a random 5 or 20% sample, we applied that fraction to our sample size counts in the comparison of sample sizes between the original and reproduction. |
| studyid | Study identifier |
| matched | Indicator of whether the numbers come from a matched population |
| original_percent_mean | Original sample size |
| reproduction_percent_mean | Reproduction sample size |
| descriptive_or_comparator | Indicator of whether the study is descriptive or comparative |
| relative_magnitude | Relative magnitude of sample size in original vs reproduction |
