# Supplementary material for: Reproducibility of real-world evidence studies using clinical practice data to inform regulatory and coverage decisions
Source: Nat Commun. 2022 Aug 31;13:5126. doi: 10.1038/s41467-022-32310-3 (PMC9430007; doi:10.1038/s41467-022-32310-3)
Supplement: Supplementary file 9 — Supplementary Dataset 6 [file 41467_2022_32310_MOESM9_ESM.zip › Supplementary Data 6 Analysis file, code, data dictionary/Installation guide.docx]

INSTALLATION GUIDE

1. Go to R website: <https://cran.r-project.org/bin/windows/base/>
2. Download latest version of R compatible with your operating system
3. Install program based on prompts from download
4. Go to the RStudio website: <https://www.rstudio.com/products/rstudio/download/>
5. Download RStudio for desktop
6. Install program based on prompts from download
7. Open a script in RStudio and install RMarkdown package using the following code: install.packages("rmarkdown")

Typical time to install <10 minutes
