# Supplementary material for: Reproducibility of real-world evidence studies using clinical practice data to inform regulatory and coverage decisions
Source: Nat Commun. 2022 Aug 31;13:5126. doi: 10.1038/s41467-022-32310-3 (PMC9430007; doi:10.1038/s41467-022-32310-3)
Supplement: Supplementary file 10 — Reporting Summary [file 41467_2022_32310_MOESM10_ESM.pdf]

nature portfolio

Corresponding author(s): Wang, Shirley

Last updated by author(s): May 26, 2022

Reporting Summary

Nature Portfolio wishes to improve the reproducibility of the work that we publish. This form provides structure for consistency and transparency in reporting. For further information on Nature Portfolio policies, see our Editorial Policies and the Editorial Policy Checklist.

Statistics

For all statistical analyses, confirm that the following items are present in the figure legend, table legend, main text, or Methods section.

n/a	Confirmed	
☐	☒	The exact sample size (n) for each experimental group/condition, given as a discrete number and unit of measurement
☒	☐	A statement on whether measurements were taken from distinct samples or whether the same sample was measured repeatedly
☐	☒	The statistical test(s) used AND whether they are one- or two-sided<br>*Only common tests should be described solely by name; describe more complex techniques in the Methods section.*
☐	☒	A description of all covariates tested
☐	☒	A description of any assumptions or corrections, such as tests of normality and adjustment for multiple comparisons
☐	☒	A full description of the statistical parameters including central tendency (e.g. means) or other basic estimates (e.g. regression coefficient) AND variation (e.g. standard deviation) or associated estimates of uncertainty (e.g. confidence intervals)
☐	☒	For null hypothesis testing, the test statistic (e.g. F, t, r) with confidence intervals, effect sizes, degrees of freedom and P value noted<br>*Give P values as exact values whenever suitable.*
☒	☐	For Bayesian analysis, information on the choice of priors and Markov chain Monte Carlo settings
☒	☐	For hierarchical and complex designs, identification of the appropriate level for tests and full reporting of outcomes
☐	☒	Estimates of effect sizes (e.g. Cohen's d, Pearson's r), indicating how they were calculated

Our web collection on statistics for biologists contains articles on many of the points above.

Software and code

Policy information about availability of computer code

Data collection	Aetion Evidence Platform® (2021) v4.2, SAS 9.4
Data analysis	Aetion Evidence Platform® (2021) v4.2, SAS 9.4, STATA 14, and Cran R version 3.6.1 (https://osf.io/my5gn/)

For manuscripts utilizing custom algorithms or software that are central to the research but not yet described in published literature, software must be made available to editors and reviewers. We strongly encourage code deposition in a community repository (e.g. GitHub). See the Nature Portfolio guidelines for submitting code & software for further information.

Data

Policy information about availability of data

All manuscripts must include a data availability statement. This statement should provide the following information, where applicable:
- Accession codes, unique identifiers, or web links for publicly available datasets
- A description of any restrictions on data availability
- For clinical datasets or third party data, please ensure that the statement adheres to our policy

The data used to generate tables and figures for this paper are available in Data S6, which has been deposited in the Open Science Framework under accession code https://osf.io/my5gn/. The raw data used to generate the study data are available under restricted access only. Members of the reproduction team were permitted to access the raw data provided by third parties. The raw data are protected and are not publicly available due to data privacy laws and data use agreements. The processed data used to generate tables and figures are available in the Open Science Framework repository (DOI 10.17605/OSF.IO/MY5GN).

Our data use agreements for MarketScan, Optum, CPRD and Medicare do not permit us to share source data or data derivatives with individuals and institutions not covered under the agreements. These data sources may be accessed by other investigators through their own data use agreements. The administrative and clinical

research databases used in the study reproductions are accessible to other researchers by contacting the data owner/vendors and acquiring data use agreements and/or data licenses. The research data and data derivatives cannot be shared outside of the terms of these agreements. It is our experience that the data vendors we used are very responsive to requests for contracting use of their patient data resources. However, the cost, timeframe, and process for completing the contract for authorized use of these data varies.

Contacts and information on how to acquire access to source data:

Medicare
resdac@umn.edu
https://resdac.org/research-identifiable-files-rif-requests

Optum Clinformatics
connected@optum.com
https://www.optum.com/business/solutions/life-sciences/real-world-data/claims-data.html

IBM MarketScan
https://www.ibm.com/products/marketscan-research-databases/databases

CPRD
rdg@cprd.com
https://www.cprd.com/research-applications

The remaining data are available within the Article or from the authors upon request.

Field-specific reporting

Please select the one below that is the best fit for your research. If you are not sure, read the appropriate sections before making your selection.

☐ Life sciences ☒ Behavioural & social sciences ☐ Ecological, evolutionary & environmental sciences

For a reference copy of the document with all sections, see nature.com/documents/nr-reporting-summary-flat.pdf

Behavioural & social sciences study design

All studies must disclose on these points even when the disclosure is negative.

Study description	Quantitative cross-sectional descriptive study of the reproducibility of 150 database studies (sampled studies used a cohort study design)
Research sample	Systematic random sample of published real-world evidence studies. The sample was restricted to studies conducted using 4 research databases because the research team was only able to license access to 4 research databases.
Sampling strategy	Systematic random sample. No power calculations. This was a descriptive study. The target sample size for this descriptive characterization of reproducibility was chosen based on estimates for the largest feasible number of studies that could be evaluated within the project time frame.
Data collection	We made secondary use of patient data that was routinely collected as part of clinical care.
Timing	June 1 2017-June 30 2021
Data exclusions	We did descriptive analyses on all data in our sample. No data were excluded.
Non-participation	There were no participants. We made secondary use of existing data.
Randomization	There was no intervention or randomization. We made secondary use of existing data.

Reporting for specific materials, systems and methods

We require information from authors about some types of materials, experimental systems and methods used in many studies. Here, indicate whether each material, system or method listed is relevant to your study. If you are not sure if a list item applies to your research, read the appropriate section before selecting a response.

Materials & experimental systems

n/a	Involved in the study	
☒	☐	Antibodies
☒	☐	Eukaryotic cell lines
☒	☐	Palaeontology and archaeology
☒	☐	Animals and other organisms
☐	☒	Human research participants
☐	☒	Clinical data
☒	☐	Dual use research of concern

Methods

n/a	Involved in the study	
☒	☐	ChIP-seq
☒	☐	Flow cytometry
☒	☐	MRI-based neuroimaging

Human research participants

Policy information about studies involving human research participants

Population characteristics	The populations involved in the 150 studies that were reproduced varied widely. In general, they were representative of the patient populations that could be covered in large national US employer based insurance claims, US Medicare population, and United Kingdom primary care patients.
Recruitment	This involved secondary use of existing data in administrative insurance claims and electronic health records. There was no recruitment of patients.
Ethics oversight	The study was approved by the Brigham and Women's Hospital Institutional Review Board

Note that full information on the approval of the study protocol must also be provided in the manuscript.

Clinical data

Policy information about clinical studies

All manuscripts should comply with the ICMJEguidelines for publication of clinical research and a completedCONSORT checklist must be included with all submissions.

Clinical trial registration	Registered on EUPAS: EUPAS19636
Study protocol	https://www.encepp.eu/encepp/viewResource.htm?id=39874
Data collection	We made secondary use of existing patient data between Jun 2017 and Jun 2021
Outcomes	This was a descriptive study. We pre-specified that we would evaluate:<br>• Descriptive frequencies of how often reporting was insufficient for specific parameters; measuring the extent to which the study team had to make assumptions about scientific decisions made by the original investigator.<br>• Standardized differences between original paper and replication for prevalence or mean of baseline characteristics, incidence rates/risks, and reported measures of association (absolute and/or relative).<br>• Calibration plot for reported measures of association between the original and replication with bars indicating width of 95% interval for each.<br>• The degree to which lack of transparency in different areas (e.g. timing of cohort entry and follow up, inclusion/exclusion criteria, algorithms to measure exposure, outcome, covariates etc.) relates to standardized differences for reported measures of association in the original versus the replication.
